# The impact of supported accommodation on health and criminal justice outcomes of people released from prison: a systematic literature review

**DOI:** 10.1186/s12954-023-00832-8

**Published:** 2023-07-21

**Authors:** Daisy Gibbs, Emily Stockings, Sarah Larney, Daniel J. Bromberg, Anthony Shakeshaft, Sara Farnbach

**Affiliations:** 1 National Drug and Alcohol Research Centre, UNSW Sydney, 22-32 King Street, Randwick, NSW 2031 Australia; 2 The Matilda Centre for Research in Mental Health and Substance Use, University of Sydney, Jane Foss Russel Building, Camperdown, NSW 2006 Australia; 3 Department of Family Medicine and Emergency Medicine, Universite de Montreal and Centre de Recherche du CHUM, Montreal, QC Canada; 4 Department of Social and Behavioral Sciences, Yale School of Public Health, Yale University, New Haven, CT 06511 USA; 5 Center for Interdisciplinary Research On AIDS, Yale University, New Haven, CT 06511 USA; 6 Poche Centre for Indigenous Health, University of Queensland, Toowong, QLD Australia

**Keywords:** Supported accommodation, Post-release accommodation, Prison, Systematic review, Recidivism, Community integration

## Abstract

**Background:**

Supported accommodation intends to address challenges arising following release from prison; however, impact of services, and of specific service components, is unclear. We describe key characteristics of supported accommodation, including program components and outcomes/impact; and distil best-evidence components.

**Methods:**

We conducted a systematic review, searching relevant databases in November 2022. Data were synthesised via effect direction plots according to the Synthesis Without Meta-analysis guidelines. We assessed study quality using the McGill Mixed Methods Appraisal Tool, and certainty in evidence using the GRADE framework.

**Results:**

Twenty-eight studies were included; predominantly cross-sectional. Program components which address life skills, vocational training, AOD use, and mental health appear to positively impact criminal justice outcomes. Criminal justice outcomes were the most commonly reported, and while we identified a reduction in parole revocations and reincarceration, outcomes were otherwise mixed. Variable design, often lacking rigour, and inconsistent outcome reporting limited assessment of these outcomes, and subsequently certainty in findings was low.

**Conclusion:**

Post-release supported accommodation may reduce parole revocations and reincarceration. Despite limitations in the literature, the findings presented herein represent current best evidence. Future studies should clearly define program components and measure their impact; use analyses which reflect the high risk of adverse outcomes, such as time-to-event analyses; and consider outcomes which reflect the range of challenges faced by people leaving prison.

*Registration*: PROSPERO registration CRD42020189821.

**Supplementary Information:**

The online version contains supplementary material available at 10.1186/s12954-023-00832-8.

## Introduction

The global prison population exceeds 11 million, with the rate of growth exceeding that of the general population [1]. Most people who are incarcerated are serving short sentences [2], or are held pre-trial [3]. As such, the number of people released from prison each year exceeds the daily average prison population.

The challenges for people after release from prison have been well documented [4–8], including barriers to housing, mental and physical health, employment, and barriers accessing alcohol and other drug (AOD) treatment and social services [5–7, 9–11]. These challenges form a synergistic relationship with one another and with the experience of incarceration; illicit drug use, mental health problems, and challenges with housing exacerbate one another [6, 12, 13], and each of these risks is increased by episodes of incarceration [6, 12, 14].

Evidence from cohort studies suggests interventions targeting post-release challenges can have a positive impact on social, health, and criminal justice trajectories [15–20]. Assisting people to obtain employment, for instance, affords individuals independence and structure, and is associated with reduced reoffending and reincarceration [15–17], while engagement with primary health care services in the first month following release from prison is associated with improved mental and physical health and AOD treatment engagement [18]. In addition to these positive impacts, there is some evidence for the way in which post-release interventions are delivered; low-intensity case management may facilitate sustained healthcare utilisation [19], for example, and personalised case management may reduce recidivism [20].

Post-release housing instability is particularly challenging because it increases the difficulty of meeting community corrections requirements and, therefore, the likelihood of parole breaches and revocations [21, 22]. Common challenges in securing housing include limited availability of housing type and location, financial hardship and compromised personal relationships [23]. Conversely, stable housing is associated with many benefits, including providing a setting from which to focus on locating employment and engaging with treatment services [24, 25]. Supported accommodation services provide temporary, transitional housing along with therapeutic programs, including in-house training or therapeutic interventions, case management, which may involve development, implementation, and review of a case plan to address social, health, and criminal justice needs and goals, and referral to community-based programs.

A 2018 scoping review sought to identify the effectiveness of post-release supported accommodation in improving criminal justice and health outcomes, including physical health, mental health, and substance use issues, and sought to identify the program components associated with positive outcomes for people released from prison [26]. The review identified inconsistent findings about the effectiveness of supported accommodation with respect to criminal justice outcomes. Further, the review did not report on the impact of supported accommodation on other outcomes. Finally, the program components likely to be most effective were not well documented.

The risk-needs-responsivity model for assessment and treatment for people who experience incarceration, includes three core principles: to match the level of service to a person’s risk of reoffending (risk), assess criminogenic needs and target them in treatment (needs), and tailoring interventions to the learning style, motivations, abilities, and strengths of clients (responsivity; [27]. Starting to identify and critically evaluate the individual components of post-release programs will be an important mechanism for improving the efficiency with which programs are able to be delivered, as services could tailor their program components to the needs of individual clients, their available resources and the specific context in which their program is delivered.

This systematic review aims to describe the key characteristics of supported accommodation services and to critique the methodological quality of studies of post-release supported accommodation and distil their current best evidence program components.

## Method

### Search strategy

We searched five electronic peer-reviewed literature databases (Scopus, Medline, Embase, PsycInfo, and Social Sciences Citation Index) on 5th May 2020, with updated searches completed on 22nd September 2021 and 9th November 2022. We used a comprehensive set of search terms pertaining to people in or leaving custody; release from custody; and supported housing, developed in consultation with a research librarian (Additional file 2: Appendix A). There were no limits on publication date, language, or country. We reviewed the references of included studies and relevant systematic reviews for additional papers or reports not identified by our searches.

The review protocol was registered on PROSPERO (CRD42020189821), and reporting is in line with the PRISMA guidelines [28].

### Screening of articles

We created a Zotero (version 6) library to catalogue studies and remove duplicates, and uploaded the remaining references to the Covidence platform [29] to complete eligibility screening. The research team had members proficient in English and French. Data sources (n = 2) in other languages were read via Google Translate.

Initial title and abstract screening was conducted by one reviewer (DG) due to resource constraints. Subsequently, full texts were independently screened for eligibility by two reviewers (any of DG, SL, DB, ES or a research assistant). Discrepancies were resolved through discussion and referral to a third reviewer as needed.

### Inclusion and exclusion criteria

For the purpose of this review, “prison” refers to all types of adult correctional institutions (i.e. prisons, jails, pre-trial detention centres). Supported accommodation refers to a temporary, transitional residence for adults recently released from prison, coupled with a therapeutic component (such as individual or group therapy, or skills-based workshops). Studies were included if they were conducted among adults aged 18 years or older who had been released from prison (no specific time frame since release was applied) and who received any form of supported accommodation. Included studies had to provide a sufficiently detailed description of the supported accommodation program components, such as details of the accommodation type, as well as the type and extent of support provided, factors targeted by services, and structure or restrictions of the program as assessed by the reviewers. We included any study design, including but not limited to evaluations of supported accommodation services and/or their program components, as well as protocols where supported accommodation and therapy was described but no outcome data were reported.

Studies were excluded if they were conducted in child and youth services or where the mean client age was < 18 years at intake, or if the service’s primary focus was on an area other than supported accommodation (e.g. residential substance use treatment or mental health services). Services that did not exclusively focus on these areas, but offered counselling or other support for substance use disorders or mental health problems as part of their program, were included.

### Data extraction and synthesis

Given the substantial variation in study and service type and outcomes measured, a meta-analysis was deemed a priori to not be appropriate, and a narrative synthesis was planned. We used the Synthesis Without Meta-analysis (SwiM) guidelines [30] to synthesise narrative data, with specific methods detailed in Additional file 2: Appendix C.

Data for each study were extracted and summarised into spreadsheets developed specifically for this review. All studies were extracted by the lead author, with 25% of studies extracted by a second person to ensure accuracy. Any discrepancies were resolved through discussion.

The characteristics of supported accommodation services are presented in Additional file 1: Table 1, including the service structure and support, program components, outcomes, measures, and impact. A list of program components delivered by each service was developed during analysis via discussions between two reviewers to ensure all program components were captured (refer to detailed definitions in Additional file 2: Appendix E). Best evidence program components were determined by identifying those components common across outcome evaluation studies, and describing the direction of these programs’ impact on client outcomes.

### Characteristics of supported accommodation for people released from prison

Data were extracted regarding nine pre-determined characteristics relating to supported accommodation: (1) year of publication; (2) country of study; (3) study type; (4) gender of clients; (5) supported accommodation type; (6) supported accommodation description (including structure, support provided, and restrictions); (7) program components; (8) outcomes/impact of program (including outcome measures used); and (9) summary of the outcomes/impact of the clients.

### Quality assessment of literature and grading of evidence

The methodological quality of studies was appraised using the McGill Mixed Methods Appraisal Tool (MMAT); [30, 31], which was developed for concurrent critical assessment of qualitative, quantitative, and mixed methods research. Study types were assessed on five relevant criteria (which vary slightly depending on study design), with response options of yes, no, or can’t tell available for each criteria. Methodological quality of studies was appraised by one reviewer, with 10% of studies reassessed by a second reviewer. Criteria for appraisal of specific study type is detailed in Additional file 2: Appendix F.

To assess our certainty of the synthesis findings, relevant components of the GRADE Framework (Grading of Recommendations, Assessment, Development and Evaluations; [32] were applied to criminal justice outcomes (rearrest, reconviction, reincarceration, parole revocations), as well as mental health and wellbeing outcomes. These components were assessment of risk of bias (ROB) at the outcome level, and the direct relationship between the intervention and outcome being assessed. To adapt MMAT findings to the ROB component of the GRADE Framework, we determined that where studies received a ‘No’ or ‘Can’t tell’ response to two domains of the MMAT, they would be considered to have some concerns with respect to ROB. Studies which received a ‘No’ or ‘Can’t tell’ response to three or more domains would be considered to have a high ROB. Two reviewers conducted independent GRADE assessments (DG, ES) with discrepancies resolved via discussion.

### Effect direction plots

Effect direction plots were used as a standardized metric to synthesise the effect measures of the included studies. This was applied to all studies which employed a comparison group, including propensity score matched, non-equivalent, and those matched against expected outcome rates. This strategy considers study design and study quality in its presentation of effect direction.

## Results

### Published studies describing supported accommodation for people released from prison

**Fig. 1:**
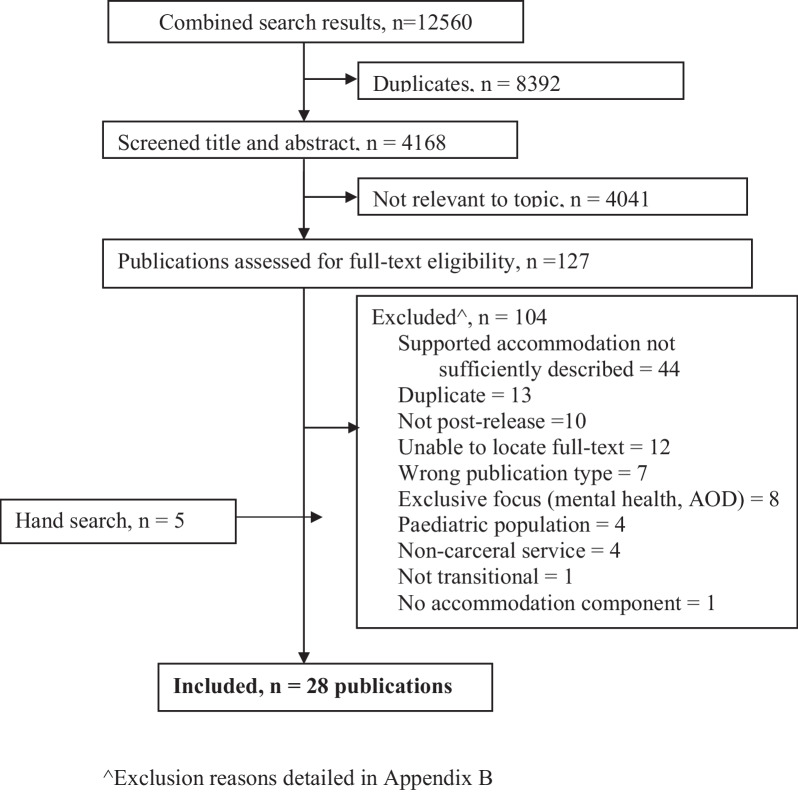
PRISMA Flowchart. ^Exclusion reasons detailed in Appendix B

We screened 4168 unique peer-reviewed publications, consisting of 4107 records identified in our search and 61 studies identified in handsearching existing relevant systematic reviews (Fig. 1). Twenty-eight studies were included in our review, describing 100 services. The list of studies excluded after full text screening and reasons for exclusion are shown in Additional file 2: Appendix B.

Study details are described in Additional file 2: Appendix D. Common study designs were cross sectional (n = 10) and cohort (n = 5). Seven studies were outcome evaluations (some studies employed multiple designs; refer to Additional file 1: Table 1). Studies were mostly conducted in the United States (n = 21), with three in the United Kingdom, two in Australia, and one New Zealand. Studies were published between 1964 and 2022, with most (n = 17) after 2010.

### Methodological quality of included studies

A summary table and further detail about methodological quality of included studies is detailed in Additional file 2: Appendix G. Only four studies satisfied all quality criteria for quantitative non-randomized studies (shaded rows in Additional file 1: Table 1; [33–36], although one study was not scrutinised by peer-review [37]. All qualitative studies met all quality appraisal criteria.

### Key characteristics of supported accommodation services

#### Service structure

Most services were classified as fixed-site accommodation with on-site support, where clients lived in one central dwelling, and support services were provided on-site [34–36, 38–49]. This also included several services wherein primary support included on-site case management, complemented by additional on-site supports and/or referral to external community-based services for further support needs (n = 19 studies; n = 72 services; [34, 36, 37, 41, 44, 45, 48–55]. Support duration, service capacity, and client gender are all described in Additional file 1: Table 1.

#### Support provided by*** services***

Details of all support provided are presented in Additional files 1 Additional file 2: Table 1. The most commonly reported form of support provided by services was case management (n = 15 studies, n = 72 services), followed by referral to community-based services (n = 52). Group and individual therapy were provided by 65 and 27 services, respectively. The therapeutic approach to case management and therapy (both individual and group) was not explicitly described in any study, although Day et al. described case managers adopting the strengths-based Good Lives Model [53, 57].

#### Program components

Program component definitions for each study are provided in Additional file 2: Appendix E. The most common program components were those that target vocational skills or employment (n = 20 studies, n = 87 services) and AOD use (n = 17 studies, n = 73 services), followed by 67 services targeting education and 66 targeting mental health and wellbeing. Fifty-six services provided life skills training and 54 provided financial training. Thirty-one services sought to address long-term housing needs (Additional file 1: Table 1)

### Impact of supported accommodation for people released from prison

Outcome measures were reported in 18 studies, most of which related to criminal justice, housing, and mental health. Only nine studies included a comparison group in their analysis and reporting [35–39, 51, 58–60], and these are synthesised below via vote counting based on direction of effect. Outcomes for all studies, including those without comparator groups, are presented in Additional file 1: Table 1.

#### Criminal justice outcomes

Six of the studies which reported criminal justice-related outcomes used quasi-experimental study design [35–38, 51, 58]. These outcomes varied, including re-arrest, reconviction, reincarceration, and parole revocation, with incidence and time-to-event measures most common amongst these. Different methods were used to match exposure and control groups, including propensity score matched comparisons [36, 50, 51, 58], and case-matched comparisons [38]. While we identified a positive effect of supported accommodation on decreasing parole revocations and reincarceration, few studies showed an unequivocal benefit of supported accommodation in terms of other criminal justice outcomes.

**Fig. 2:**
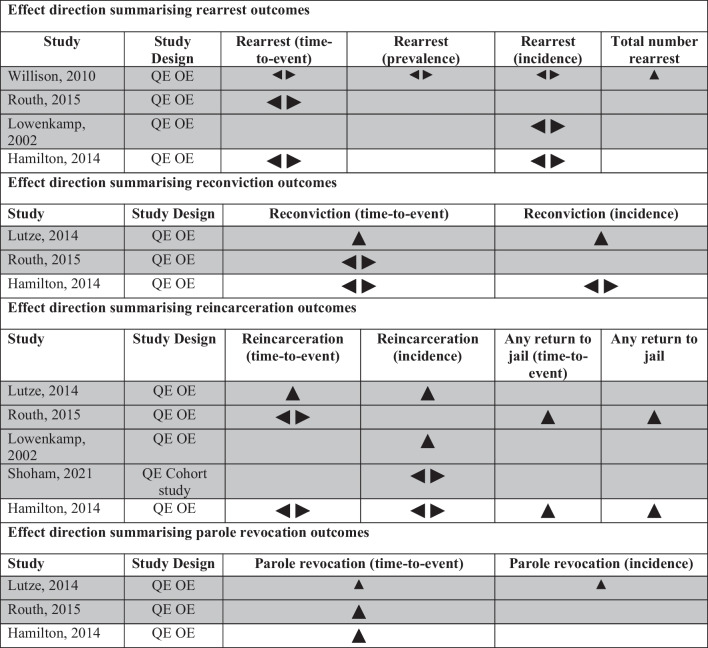
Effect direction summarising criminal justice related outcomes. Study design: QE: Quasi-experimental; OE: Outcome evaluation. Effect direction: upward arrow ▲= positive impact, downward arrow ▼= negative impact, sideways arrow ◄►= no change/mixed effects/conflicting findings. Sample size: Final sample size (individuals) in intervention group Large arrow ▲>300; medium arrow ▲ 50-300; small arrow ▲ <50. Study quality: denoted by row colour: grey = low risk of bias; white = some concerns

*Rearrest* Four studies reported rearrest outcomes using a matched comparator [37, 38, 51, 58]. One of the four included studies reported a positive rearrest outcome with a lower number of rearrests amongst treatment group versus control [37]. This same study reported mixed results for time to rearrest, with a shorter time to rearrest amongst the treatment group than control. The remaining studies found no between group difference in time to rearrest, incidence, or prevalence rearrest. See Fig. 2.

*Reconviction* Two reconviction outcomes were reported across four studies, three of which had a propensity score matched comparator [35, 51, 58] and were included in the vote counting synthesis, see Fig. 2. One of the three included studies reported positive reconviction outcomes including a significantly longer time to new conviction for program clients and significantly fewer new convictions in the study period [35]. The remaining two studies reported no difference in reconviction outcomes, including time to reconviction and reconviction incidence between treatment and control groups.

*Reincarceration* Five studies reported reincarceration outcomes [35, 36, 38, 51, 58], all of which employed matched comparators, of which four were propensity score matched, and all five studies were included in synthesis. Four of the five studies reported positive reincarceration outcomes, including reduced reincarceration incidence [35, 38] for supported accommodation clients versus control, as well as longer time to reincarceration for any reason, including parole violations [51, 58], and significantly reduced incidence of reincarceration for any reason [51]. Shoham et al. reported increased reincarceration when compared with the control group (2021). Time to reincarceration results were mixed, with one study reporting longer time to reincarceration for clients [35], while two others reported no difference between treatment and control groups [51, 58].

*Parole revocations* Three studies reported parole revocation outcomes [35, 51, 58]. All three studies found a longer time to parole revocation for program clients than the propensity score matched control, a finding which was statistically significant in two of three studies [35, 51]. Lutze et al. also found fewer revocation events in the study period than the comparator (2014).

#### Housing outcomes

Three studies examined housing outcomes for clients of supported accommodation services [35, 52, 59], one of which included a propensity score matched comparison group [35]. Periods of homelessness and number of address changes in a specific period were both reported in two studies, both with mixed results [35, 52]. Pleggenkuhle et al. reported clients in supported accommodation had fewer problems with their current residential situation than the control group. Program clients also reported great satisfaction with their residential situation, and an increased feeling of autonomy [59]. GRADE rating indicated low certainty in these findings, and caution should be taken with interpretation.

#### Other social and mental wellbeing outcomes

Six studies reported other social and mental wellbeing outcomes [41, 44, 49, 52, 54, 59, 61]. One included a control group and reported a positive effect direction on feelings of autonomy [59], however the data were qualitative and like the remaining studies, used self-reported outcomes [41, 44, 49, 54]. Other outcomes included community integration, sense of autonomy or control over one’s life, stigma, cultural connectedness, and a number of mental health outcomes. See Additional file 1: Table 1 for narrative synthesis of outcomes. Due to the variation in study design, small number of studies to each outcome, lack of appropriate control groups, and often ill-defined nature of outcomes and their measures, our confidence in these findings is limited and caution should be taken when interpreting these findings.

#### Impact of specific program components

One study included in the review examined the impact of different program components on rearrest outcomes [37]. Components which targeted AOD use, specific criminal behaviour, and life skills were found to have no significant effect on rearrest, while spirituality and faith based components were found to be associated with an increase in rearrest.

#### Best evidence program components

Seven studies were outcome evaluations [35–39, 58, 60], and due to the lack of outcome information related to specific program components, we pooled these evaluations to identify the impact of certain program components on client outcomes. Across these evaluations, there was no one program component common to all studies. Vocational training and employment skills, AOD use, mental health and wellbeing, and life skills components were the most commonly reported, each described in four of the seven studies. Consistent with the above results, GRADE rating indicated low certainty in these findings, and caution should be used in interpretation.

The four studies describing vocational skills and employment training included matched comparison groups, two of which were propensity score matched [51, 58]. The studies reported mixed effect directions for rearrest [37, 38, 51], reconviction [51, 58], and reincarceration [38, 51, 58] outcomes. Both studies reporting on the outcome described a positive effect direction for time to parole revocation, and return to jail for any reason, the latter being significant in both studies [51, 58].

The same four studies reported on program components targeting AOD use and mental health and wellbeing. One study employed propensity score matching and reported a positive effect on any return to jail and parole revocations [51]. Three studies described mixed effect on new convictions [51] and rearrest [37, 38, 51], these employed a range of comparison groups including propensity score matching and a matched group of expected rates [60]. Willison et al. provided the only study to examine the impact of different program components on arrest incidence and prevalence, with no significant between-group difference detected for those who participated in AOD use programs (2010).

Four studies described program components that targeted life skills, three of which used matched control groups [35, 37, 38]. These studies reported positive effect on reincarceration outcomes, including longer time to reincarceration and reduced prevalence of reincarceration [35], both of which were significant findings. Two studies reported a longer time to parole revocation [35, 58], while there was no between-group difference detected for percent rearrested [37, 38].

## Discussion

Our systematic review of literature describing supported accommodation for people released from prison identified considerable variability in how these services operate and are evaluated. Data derived from 28 studies indicated improvement in key domains, such as parole revocations and reincarceration. Despite limitations, including low certainty in evidence due to observational nature of literature and inclusion of few studies in synthesis, the current review represents the best available evidence. Detailing the program components and operation of supported accommodation services, and synthesising the impact of these program components, our review extends current research beyond a 2018 scoping review of the supported accommodation literature [26], and herein we discuss explicit recommendations for further improvement to the evidence base. We recommend that future studies of supported accommodation clearly define and measure the impact of program components; use outcome measures which accurately reflect the high risk of adverse outcomes, such as time-to-event analyses; and study a range of outcomes, not exclusively those related to criminal justice.

Our review found limited evidence for the impact of specific program components on client outcomes. Few studies reported outcomes which related to the many non-criminal justice related challenges experienced following release from prison, including housing and mental and physical health. To improve the standard and scalability of supported accommodation services for people released from prison, we recommend that more resources be allocated to clearly documenting program components, as well as evaluations of their impact on a range of outcomes spanning criminal justice, health, and social wellbeing.

Although only one study reported on the impact of individual program components on client outcomes, the impact of certain components may be inferred by considering client outcomes reported in outcome evaluation studies with common components. Across these seven studies vocational training and employment skills [37, 38, 51, 58], AOD use [37, 38, 51, 60], mental health and wellbeing [37, 38, 51, 60], and life skills [35, 37–39], were each common to four studies. Life skills programs appear to have a positive impact on criminal justice outcomes, as do vocational training, AOD use, and mental health programs. While caution should be taken due to limitations related to the individual studies, and the potential to interpret outcomes collectively, these results represent the current best available evidence.

Most studies provided limited details regarding program components, and simply named these without clear definition (see Additional file 2: Appendix E for program component definitions or description). At present the academic literature gives little indication that program components of supported accommodation have been standardised or manualised, which has implications for training and scaling up programs, as well as standardising programs across services where there is evidence of their effectiveness. It is recommended that authors provide, at minimum, information about the length of stay of clients and service capacity. We also recommend that program components be clearly defined and ideally manualised with detail including the therapeutic approach to program delivery. Detailing program components will improve opportunities for ongoing monitoring and evaluation, as well as standardisation and scale up.

Although not specifically an outcome measure of this review, studies did not describe the mechanism of change for program components, the operational process by which targeted outcomes are thought to be achieved. Including this detail is recommended as it could improve service provision by more clearly defining how program components are expected to impact on a client; greater clarity regarding program components and articulation of how they achieve targeted outcomes. This information would allow services to design and adapt their programs to client needs and available resources, based on best evidence. Consultation with supported accommodation service providers, along with embedding ongoing data collection processes into routine service delivery, may also elucidate more details about the function of program components in achieving client outcomes [62].

We identified weak evidence for a positive impact of supported accommodation on criminal justice outcomes. It is important to bear in mind, however, that clients of supported accommodation services are potentially more closely supervised than comparison groups, with parole violations and offending more likely to be detected. Further, eligibility for services is often limited to those who are at greater risk of re-offending which may skew outcomes towards higher rates of criminal behaviour due to the higher risk of participants at study outset [35, 36, 40, 50, 51, 61]. Although a number of studies identified in our review employed propensity score matching for comparison groups, meaning that this increased risk is reflected in both study arms, criminal justice outcomes should be based on time-to-event analysis to determine the impact of service attendance and reflect this high likelihood of reincarceration.

Criminal justice related outcomes were the most commonly reported amongst the included studies, and our review identified a positive effect of supported accommodation on parole revocations and reincarceration outcomes. However, the nearly exclusive use of criminal justice outcomes to measure the impact of supported accommodation overlooks the complex challenges experienced by people who are released from prison. Such a focus contributes to a deficit narrative which does not account for the broad range of factors which contribute to the likelihood of becoming (re)incarcerated, including sociodemographic and health factors such as housing [63], substance use [64–66], mental health [66], and physical health [5, 6, 18, 67–70]; as well as systemic issues such as the over policing of racial and ethnic minority communities [71, 72] and the intimate relationships between the carceral system and other state infrastructure such as schools, hospitals, and community mental health services [73–75]. By targeting a range of these challenges, supported accommodation services have the potential to address outcomes other than those related to reoffending or recidivism. We recommend that future studies extend the evidence beyond criminal justice measures by measuring the impact of these services on non-criminal justice outcomes which reflect the challenges faced by this population. Engaging service providers in the research design process may increase the opportunity to identify a broader range of outcomes and outcome measures which reflect the range of challenges addressed through supported accommodation.

In the context of such a broad range of known challenges experienced by people released from prison, which contribute to the risk of reoffending, and the wide range of factors targeted by supported accommodation services, detailed risk assessment which incorporates identification of client needs is important to ensure appropriate service delivery. While some of the included studies described program eligibility criteria which included risk assessments [35, 40, 50, 51, 58, 59, 61], most studies either had criteria that were not specific to the client risk level, or did not report eligibility criteria at all. Three studies described services which applied, to varying degrees, the risk-needs-responsivity model [40, 53, 56], which argues that interventions for people who experience incarceration should be tailored to a person’s capacity and informed by their risk of reoffending specific and criminogenic need [27]. More consistent application of this model to delivery of supported accommodation services for people leaving prison may ensure effective intervention delivery.

Further to the already described challenges related to scalability and replicability, the lack of clear program component definitions or evidence of standardisation in the academic literature creates challenges in understanding how program components can be delivered in a responsive manner. This includes targeting and dosing appropriate program components according to client risk and needs, as well as their strengths and motivations. Embedding data collection processes in service delivery may increase the opportunity to identify client risk level and needs, as well as identifying dose information. This could also contribute to measurements of the impact of certain program component on specific outcomes.

## Limitations of the literature

Only 12 of the 28 included studies met all methodological quality criteria (Additional file 2: Appendix G), and it is important to note that the nature of our review included studies which did not exclusively evaluate supported accommodation for people released from prison. While studies may have been methodologically satisfactory, our review also includes studies which only describe supported accommodation, in addition to those which evaluated these services.

Overall, while the included studies do not present overt risk of bias at the outcome level, a small number of studies were included in syntheses, and there is a lack of rigorous study designs that permit causal inference regarding supported accommodation effectiveness. Further, certain outcomes included sub-analyses affected by selection bias (e.g. outcomes for “program completers” compared to non-program participants) resulting in mixed effect directions. Program evaluations need to be undertaken to a higher standard to allow for better understanding of effectiveness. As a result, we are limited to the information that was reported by the authors in the included literature regarding program components of these services, and work remains to identify the impact of specific components on specific outcomes. While randomized control trials of supported accommodation for people released from prison may not be feasible due to ethical considerations regarding assigning individuals to substandard housing conditions when at risk of homelessness, novel approaches to evaluation design may be employed to improve the quality of this evidence. Future research could usefully integrate multiple data sources including linked administrative data with expertise of service providers and clients to identify program components and their impact on client outcomes.

Many studies evaluated outcomes across multiple facilities, without clarification around the consistency of intervention delivery. This, coupled with varied detail provided describing supported accommodation services and their program components, and varied study types created challenges when analysing data in this review. Further, we were limited in our scope to compare the effectiveness of interventions because of the above-mentioned inconsistent definitions as well as the variation in how these program components were combined in service delivery.

Finally, most studies identified in our review were drawn from the United States (Additional file 2: Appendix D) which presents a specific criminal justice and cultural context. There is a need for more research from other countries.

## Limitations of this review

Given the fragmented nature of the literature, we were unable to draw unambiguous conclusions regarding the effectiveness of supported accommodation services following release from prison on either legal or health outcomes. Relatedly, we were unable to identify specific program components consistently linked with better client outcomes. Both these limitations could be addressed through primary research studies that adopt rigorous methods, clearly document program components, and explore important health as well as legal outcomes. Finally, having only one reviewer screening articles at the title and abstract stage may have had consequences for screening consistency and increasing possible selection bias.

## Conclusion

People who are released from prison experience social, financial, and health challenges; supported accommodation aims to help improve outcomes during the post-release period. This review represents the current best available evidence regarding the key characteristics of these services, however we identified considerable variability in how supported accommodation services operate and are evaluated. We found a positive effect of supported accommodation on reincarceration and parole revocation outcomes, although other outcomes had mixed direction of effect. Vocational training and employment skills and life skills program components may have a positive effect on parole revocations and reincarceration outcomes, however these findings should be interpreted with caution. We recommend that future research evaluates the impact of specific program components on client outcomes, and that studies consider outcomes which reflect to the range of challenges experienced following release from prison, including housing, mental and physical health.

## Supplementary Information

**Additional file 1.** Table 1: Key characteristics of identified services, and their effectiveness.

**Additional file 2.** Supported accommodation: A systematic review: Appendices.

## Data Availability

Our review was registered with PROSPERO (CRD42020189821), and our search terms were developed in consultation with a research librarian can be found in Appendix A.

## Abbreviations

AOD: Alcohol and other drugs
GRADE: Grading of recommendations, assessment, development and evaluations
MMAT: Mixed methods appraisal tool
ROB: Risk of Bias
RNR: Risk needs responsivity
