# Supplementary material for: The impact of supported accommodation on health and criminal justice outcomes of people released from prison: a systematic literature review
Source: Harm Reduct J. 2023 Jul 21;20:91. doi: 10.1186/s12954-023-00832-8 (PMC10362610; doi:10.1186/s12954-023-00832-8)
Supplement: Supplementary file 1 — Additional file 1. Table 1: Key characteristics of identified services, and their effectiveness. [file 12954_2023_832_MOESM1_ESM.docx]

## Table 1: Key characteristics of identified services, and their effectiveness

|  |  |  | **Program characteristics** | | **Program outcome/impact** | |
| --- | --- | --- | --- | --- | --- | --- |
| **Author & year of publication (country)** | **Study design*^a^*** | **Facilities (n)** | ***Supported accommodation type***  **Supported accommodation description** | **Program components** | **Outcomes/ impacts of program**  **(Outcome measure)** | **Summary of outcomes/impacts of program on clients**  (**bold**) indicated matched comparator |
| **Simonds, 2022 (USA)** | RCT | *1* | *Multi-site*  **Structure: 3**-month accommodation for men. Service capacity not described.  **Support:** CM; referral to community-based services.  **Restrictions:** Not described. | 1. Education 2. Vocational skills & employment 3. AOD use 4. Life skills 5. Financial skills | Not reported | Not reported |
| **Willison, 2010 (USA)** | CBA | *1* | *Multi-site*  **Structure:** 90-day program. 6 single family homes located in residential neighbourhoods with capacity ranging from 6-8 beds in each house, total 38 beds.  **Support:** CM; individual and group therapy; referral to community-based services; peer-to-peer support.  **Restrictions:** Engagement with fulltime employment, sobriety, random drug testing. | 1. Permanent housing 2. Vocational skills & employment 3. AOD use^e^ 4. Mental health and wellbeing, including anger management 5. Physical health 6. Life skills ^e^ 7. Financial skills 8. Spirituality & faith connections ^e^ 9. Connections to family & community ^e^ 10. Self-efficacy/self-esteem 11. Specific criminal behaviour ^e^ 12. Domestic violence | Rearrest (event incidence and prevalence and time-to-event; AD) | ≈ No between-groups difference in percentage rearrest ^f^  ≈ No between-groups difference in incidence rearrest ^f^  **↓ Lower number of total rearrests** ^f^  **↓ Shorter time to rearrest *** ^f^  ↑ Longer time to rearrest than control group amongst those who completed treatment* |
| **Dowell, 1985 (USA)** | CBA | *1* | *Fixed-site*  **Structure:** Bed and board to 6-12 women at a time. Length of stay not described.  **Support:** Inconsistent support during period of observation. Intermittent counselling and “rap groups”  **Restrictions:** Not reported | 1. Life skills | Reoffending (Frequency; AD)  Severity of offense (US Bureau of Prisons index score; AD) | ↓ Average number of crimes half that of comparison group  ↓ Average severity of crime for which treatment group were arrested for less than two-thirds severity of control group |
| **Routh, 2015 (USA)** | PCS | *12* | *Multi-site*  **Structure:** Not detailed.  **Support:** Group programs; referral to community-based services.  **Restrictions:** Not detailed. | 1. Vocational skills & employment 2. Connections to family & community | Rearrest (Time-to-event; AD)  Reconviction (Time -to-event; AD)  Reincarceartion (Time -to-event; AD)  Parole revocation due to technical violation (Event and time-to-event; AD)  Any return to jail (Time -to-event; AD) | ≈ No between-groups difference in time to rearrest  ≈ No between-groups difference in time to reconviction  ≈ No between-groups difference in time to reincarceration  **↑ Longer time to revocation**  **↑ Longer time to return to jail for any reason*** |
| **Hamilton, 2014 (USA)** | PCS | *18* | *Variable dependent on setting and client need*  **Structure:** Service for men. Capacity and accommodation duration not detailed.  **Support:** CM; individual and group therapy; on-site training.  **Restrictions:** Random drug screening | 1. Education 2. Vocation skills & employment 3. AOD use 4. Mental health and wellbeing, including anger management | Rearrest (IR and time-to-event; AD)  Reconviction (IR and time-to-event; AD)  Reincarceration for new offense (IR and time-to-event; AD)  Parole revocation (IR and time-to-event; AD)  Any return to jail (IR and time-to-event; AD) | **↑ Longer time to parole revocation ***  **↑ Longer time to any return to jail ***  **↓ Reduced incidence of return to jail for any reason***  **≈ No difference in rearrest**  **≈ No difference in time to rearrest**  **≈ No difference in reconviction**  **≈ No difference in time to reincarceration** |
| **Lutze, 2014 (USA)**^b^ | PCS | *3* | *Fixed-site*  **Structure:** 12-month residential support for women. Capacity not detailed.  **Support:** Case management (CM); referrals to community-based services.  **Restrictions:** Not reported | 1. Life skills | Stable housing (identified valid address; AD^c^)  Reconvictions (IR^d^ and time-to-event; AD)  Reincarceration (IR and time-to-event; AD)  Parole revocation (IR and time-to-event; AD) | **↑ Longer time to reconviction***  **↓ Fewer reconvictions***  **↑ Longer time to parole revocation***  **↓ Fewer revocation events**  **↑ Longer time to***  **↓ Fewer reincarcerations***  **↓ Fewer subjects experienced one or more periods of homelessness***  **≈ Comparable number of address changes** |
| **Lowenkamp, 2002 (USA)**  **Men’s HWH (Halfway house) Programs** | PCS | *21* | *Fixed-site*  **Structure:** The average capacity of the Men’s HWH programs is 48 (range: 15-105) with an average length of stay of 95 days (range: 53-120 days).  **Support:** CM, group therapy; on-site training for specific modules; group programs.  **Restrictions**: Two programs exclude: people charged with arson or sexual crimes. One program excludes those with mental and physical disabilities. | 1. Permanent housing (1/21 programs) 2. Education (13/21 programs) 3. Vocation skills & employment (17/21 programs) 4. AOD use (20/21 programs) 5. Mental health and wellbeing, including anger management (14/21 programs) 6. Life skills (16/21 programs) 7. Financial skills (15/21 programs) 8. Self-esteem/efficacy (2/21 programs) 9. Spirituality & faith connections (6/21 programs) 10. Specific criminal behaviour (3/21 programs) 11. Domestic violence (2/21 programs) | Outcomes reported for all services  Rearrest (IR; AD)  Reincarceration for technical violation (IR; AD)  Reincarceration for new offence (IR; AD)  Any reincarceration (IR; AD) | Outcomes reported for all services  **≈ No between group difference in rearrest**  **↓ Reincarceration due to technical violations lower amongst residents**  **↓ Reincarceration due to new offences lower amongst residents**  **↓ Reincarceration due to any reason lower amongst residents** |
| **Lowenkamp, 2002 (USA)**  **Women’s HWH Programs** |  | *9* | *Fixed-site*  **Structure:** The average capacity of the Women’s HWH programs is 40 (range: 19-100) with an average length of stay of 80 days (range: 58-102).  **Support:** CM, group therapy; on-site training for specific modules; group programs.  **Restrictions**: One program excludes people charged with arson or sexual crimes. | 1. Education (7/9 programs) 2. Vocation skills & employment (8/9 programs) 3. AOD use (8/9 programs) 4. Mental health and wellbeing, including anger management (7/9 programs) 5. Life skills (7/9 programs) 6. Financial skills (6/9 programs) 7. Spirituality & faith connections (2/9 programs) 8. Self-esteem/efficacy (1/9 programs) 9. Domestic violence (4/9 programs) 10. Gender needs (5/9 programs) |  |  |
| **Lowenkamp, 2002 (USA)**  **Men’s and Women’s (mixed) HWH Programs** |  | *7* | *Fixed-site*  **Structure:** The average capacity of the mixed Men’s and Women’s HWH programs is 90 (range: 16-140) with an average length of stay of 100 days (range: 89-120 days).  **Support:** CM, group therapy; on-site training for specific modules; group programs.  **Restrictions**: One program excludes people charged with arson, sexual crimes, or extremely violent crimes. | 1. Education (4/7 programs) 2. Vocation skills & employment (6/7 programs) 3. AOD use (7/7 programs) 4. Mental health and wellbeing, including anger management (3/7 programs) 5. Life skills (6/7 programs) 6. Specific criminal behaviour (2/7 programs) 7. Financial skills (5/7 programs) 8. Self-esteem/efficacy (1/7 programs) 9. Spirituality & faith connections (5/7 programs) |  |  |
| **Shoham, 2021 (Israel)** | RCS | *1* | *Fixed-site*  **Structure:** 9 months accommodation for men, followed by 1 year follow-up. 11-14 bed capacity.  **Support:** Individual and group therapy; on-site training.  **Restrictions:** AOD abstinence; frequent urine testing. | 1. Vocational skills & employment 2. AOD use 3. Life skills 4. Finances 5. Connections to family & community | Reincarceration (rate, risk, and cumulative probability; AD) | **↑ Significantly high reincarceration rate amongst all treatment participants**  **↓ Lower reincarceration rate amongst treatment completor group**  **↓ Consistently lower risk of reincarceration at the end of each year for 5 years following release amongst treatment completor group**  **↓ Consistently lower cumulative probability of returning to prison at end of each year for 4 years following release amongst treatment completor group** |
| **Davies, 2018 (UK)** | CSS | *4* | *Fixed-site*  **Structure:** 10-16 weeks accommodation for men, 24-26 bed capacity.  **Support:** 24-hour staffing; daily meetings.  **Restrictions:** Not reported | Not described | Social climate (Essen Climate Evaluation Schema; self-report)  Happiness (self-report, Oxford Happiness Questionnaire – Short; self-report)  Satisfaction with one’s life (Satisfaction with Life Scale; self-report)  Interpersonal relationship characteristics (Inventory of Interpersonal Problems; self-report)  Problem solving (Social Problem-Solving Inventory – Revised; self-report) | ↓ Residents scored lower on satisfaction with life than staff  ↓ Residents scored lower on happiness than staff  ↑ Residents scored higher on experiencing safety than staff  ↑ Residents rated the climate of Aps higher than those in other rehabilitative custodial setting |
| **Hignite, 2017 (USA)** | CSS | *1* | *Independent supported living*  **Structure:** Rapid post-release housing for men. Length of accommodation and service capacity not detailed.  **Support:** CM; individual therapy; referral to community-based services.  **Restrictions:** Not described | 1. Long-term housing 2. Education 3. Vocation skills & employment 4. AOD use ^g^ 5. Mental health and wellbeing including anger management ^g^ 6. Financial skills 7. Life skills 8. Domestic violence ^g^ | Housing (Program data)  Mental health needs (Adult-TRAG^x^), including risk of harm to oneself and/or others, support needs, employment problems, housing instability, substance use issues, criminal justice involvement, psychiatric-related hospitalisation and functional impairment | 46% secured permanent housing  ↓ Significant decrease in risk of harm to oneself or others  ↓ Significant decrease in support needs, indicating increased available supports (family, friends, community resources)  ↓ Significant decrease in employment problems  ↓ Significant decrease in housing instability  ↓ Significant decrease in cooccurring substance use issues  ↓ Significant decrease in criminal justice involvement  ≈ No client data on hospitalisations  ≈ No change in functional impairment |
| **Vigesaa, 2016 (USA)** | CSS | *1* | *Fixed-site*  **Structure:** Service for women. Length of stay or capacity not reported.  **Support:** Individual and group therapy; on-site training.  **Restrictions:** Residents are allowed to leave to work and educational opportunities. | 1. Life skills 2. Financial skills | Not reported | Not reported |
| **Morley, 2014 (USA)** | CSS | *1* | *Fixed-site*  **Structure:** 40 bed capacity for men. Length of stay and staffing information not available.  **Support:** CM; individual and group therapy; referral to community-based services; on-site training.  **Restrictions:** Not reported | 1. Education ^g^ 2. Vocational skills & employment 3. AOD use 4. Mental health and wellbeing including anger management 5. Life skills 6. Connections to family & community | Recidivism (IR; AD) | 15% recidivism rate for those who complete the program |
| **Ellison, 2013 (UK)** | CSS | *1* | *Scattered-site*  **Structure:** Length of accommodation and capacity not described.  **Support:** Referral to community-based services.  **Restrictions:** Monthly property inspections. | 1. AOD use ^g^ 2. Mental health and wellbeing including anger management ^g^ 3. Physical health ^g^ 4. Financial skills ^g^ | Reoffending (IR; AD) | ↓ Reduced proven re-offending rate compared to predictor of re-offending based on static risks  ↓ 11% reduction in one-year re-offending rates (for those housed)  ↓ 11% reduction in two-year re-offending rates  ↓ 21% reduction in re-offending for more serious offences  ↓ 20.9% reduction in serious offences |
| **Yeboah, 2000 (New Zealand)**  **Salisbury Street Foundation (SSF)** | CSS | *1* | *Fixed-site*  **Structure:** 12-month accommodation for men, 10-person capacity.  **Support:** Group therapy; referral to community-based services; on-site training.  **Restrictions:** Not reported | 1. Education 2. AOD use ^g^ 3. Mental health and wellbeing including anger management 4. Physical health 5. Life-skills 6. Financial skills 7. Self-esteem/self-efficacy 8. Connections to family & community 9. First Nations’ cultural connections | Community reintegration (self-reported assessment of causes of offending and reintegration)  Reconviction (IR; AD)  Cultural connection (Self report) | ↑ Assisted clients to reintegrate  ↑ Met gender needs  ↑ Clients reported satisfaction with Maori language and customs components of program  ≈ Completion rates low  ↓ Reduced reconviction compared to those on periodic detention, probration, or general reconviction |
| **Yeboah, 2000 (New Zealand)**  **Aspell House (AH)** | CSS | *1* | *Fixed-site*  **Structure:** 12-month accommodation for women, 10-person capacity.  **Support:** Group therapy; referral to community-based services; on-site training.  **Restrictions:** Not reported | 1. Education 2. AOD use 3. Mental health and wellbeing including anger management 4. Physical health 5. Life-skills 6. Financial skills 7. Self-esteem/self-efficacy 8. Connections to family & community 9. First Nations’ cultural connections 10. Gender needs | Community reintegration (self-reported assessment of causes of offending and reintegration)  Cultural connection (Self report) | ↑ Assisted clients to reintegrate  ↑ Met gender needs  ↑ Clients reported satisfaction with Maori language and customs components of program  ≈ Completion rates low |
| **Hartman, 1994 (USA)** | CSS | *1* | *Fixed-site*  **Structure:** ~20 weeks accommodation for men. Service capacity not described.  **Support:** Referral to community-based services; on-site training.  **Restrictions:** Curfews and AOD abstinence; routine AOD testing. | 1. Vocational skills & employment ^g^ 2. AOD use ^g^ 3. Life skills 4. Financial skills | Rearrest (IR and frequency; AD) | 60% felony rearrest  67% criminal rearrests |
| **Calathes, 1991 (USA)** | CSS | *1* | *Fixed-site*  **Structure:** 6-month maximum residential stage, 6-month aftercare follow-up for women. 24 bed capacity.  **Support:** Individual therapy; referral to community-based services; on-site training.  **Restrictions:** Not reported | 1. Permanent housing 2. Education 3. Vocational skills & employment 4. AOD use 5. Financial skills 6. Self-esteem/self-efficacy | Not reported | Not reported |
| **Walsh, 1990 (USA)** | CSS | *1* | *Fixed-site*  **Structure:** 4 house staff. 12 months accommodation for men.  **Support:** CM; referral to community-based services; advocacy; on-site training.  **Restrictions:** Not detailed. | 1. Education 2. Vocational skills & employment 3. Mental health and wellbeing including anger management ^g^ 4. Physical health ^g^ 5. Financial skills | Not reported | Not reported |
| **Donnelly, 1984 (USA)** | CSS |  | *Fixed-site*  **Structure:** 22-bed capacity for men. Length of stay not detailed.  **Support:** CM; referral to community-based services  **Restrictions:** Not reported | 1. Permanent housing 2. Vocational skills & employment 3. AOD use ^g^ 4. Mental health and wellbeing including anger management ^g^ 5. Financial skills 6. Connections to family & community | Not reported | Not reported |
| **Twill, 1998 (USA)** | BAC | *1* | *Fixed-site*  **Structure:** 33-120 days accommodation for men. 10 units with 36 bed capacity. 24-hour supervision.  **Support:** Individual therapy; referral to community services.  **Restrictions:** AOD abstinence; seeking and finding employment within 2 weeks; reporting back to halfway house as determined by relevant furlough and work passes; and administrative rules. | 1. Education 2. Vocational skills & employment 3. Connections to family & community | Belief in personal control (Belief in Personal Control Scale; self-report)  Social loneliness (Emotional/Social Loneliness Inventory; self-report)  Social supportive behaviours (Inventory of Socially Supportive Behaviours; self-report) | ↓ Decrease in loneliness at completion*  ↑ Increase in internal locus of control at completion* |
| **Techagaisiyavanit, 2021 (Thailand)** | MM | *1* | *Fixed-site*  **Structure:** Service for men. Capacity and length of stay not detailed.  **Support:** Collaboration with and referral to community services; individual and group therapy.  **Restrictions:** Not detailed. | 1. Vocational skills & employment 2. AOD use 3. Life skills 4. Self-esteem/self-efficacy 5. Spirituality & faith connections 6. Connections to family & community | Sense of community and social acceptance (self-report) | Not reported |
| **Williams, 2003 (USA)** | MM | *1* | *HWH*  **Structure:** Average 13 months accommodation for men. Capacity information not detailed.  **Support:** Individual and group therapy; on-site training.  **Restrictions:** Reside at facility, but employed in community. Under the direction of the court system. | 1. Vocational skills & employment 2. Mental health and wellbeing including anger management 3. Specific criminal behaviour | Quality of Life  (Qualitative open-ended questions, and Quality of Life Questionnaire; self-report) | Not reported |
| **Pleggenkuhle, 2016**  **(USA)** | QPE | *1* | *Multi-site independent*  **Structure:** 12-month housing support for men, 3-months of which is fully funded followed by stepped-down rental coverage to be taken over entirely by tenant. Approximate 30-person capacity.  **Support:** CM; group therapy; referral to community-based services.  **Restrictions:** Not reported | 1. Permanent housing 2. Life skills 3. Financial skills 4. Self-efficacy/self-esteem | Housing stability and safety (Self-report)  Social support (Self-report)  Personal agency and attitude change (Self-report) | **↓ Fewer problems with current residential situation**  **↑ Greater satisfaction with residential situation**  **≈ No between group difference in perception that housing non-permanent and experience of obstacles to finding reliable housing**  **↑ Increased feeling of autonomy** |
| **Birgerson, 2022 (Australia)** | MCS | *1* | *Multi-site*  **Structure: 12**-month accommodation for men and women. 16-person capacity.  **Support:** CM; referral to community-based services.  **Restrictions:** Not described. | 1. Education 2. Vocational skills & employment 3. AOD use 4. Mental health and wellbeing including anger management 5. Life skills 6. Financial skills 7. Self-esteem/efficacy | Not reported | Not reported |
| **Tarpey, 2016**  **(UK)** | MCS | *1* | *Within large housing association*  **Structure:** 8-24 months support. Capacity information not detailed.  **Support:** Case management; referral to community services.  **Restrictions:** Not detailed | 1. Permanent housing 2. Vocational skills & employment 3. Life skills 4. Financial skills 5. Connections to family & community | Community reintegration (self-report) | Self-reported motivation to change and reintegrate into society  Self-reported motivation in goal setting and achievement |
| **Day, 2011 (Australia)** | MCS | *1* | *Scattered-site*  **Structure:** Up to 6 months accommodation for men. 7 clients currently supported.  **Support:** 24-hour staffing; joint case management with mental health and drug and alcohol services; collaboration with and referral to community services.  **Restrictions:** Not reported | 1. Permanent housing 2. Education 3. Vocational skills & employment 4. AOD use 5. Life skills 6. Financial skills 7. Connections to family & community | Not reported | Not reported |
| **Lutze, 2009 (USA)^b^**  **Clarke** | Report | *1* | *Scattered-site housing/apartment*  **Structure:**  12 months financial housing support for men. 46-person capacity.  **Support:** CM; referral to community-based services; peer-to-peer support.  **Restrictions:** Daily reporting. | 1. Education 2. Vocational skills & employment ^g^ 3. AOD use ^g^ 4. Mental health and wellbeing including anger management ^g^ 5. Physical health ^g^ 6. Life skills ^g^ | Not reported | Not reported |
| **Lutze, 2009 (USA)^b^**  **King** | Report | *1* | *Scattered-site housing/apartment*  **Structure:**  12 months financial housing support for men. 75-person capacity.  **Support:** CM; referral to community-based services; peer-to-peer support.  **Restrictions:** Daily reporting. | 1. Education 2. Vocational skills & employment ^g^ 3. AOD use ^g^ 4. Mental health and wellbeing including anger management ^g^ 5. Life skills ^g^ 6. Connections to family & community 7. Specific criminal behaviour ^g^ 8. Domestic violence ^g^ | Not reported | Not reported |
| **Lutze, 2009 (USA)^b^**  **Spokane** | Report | *1* | *Scattered-site housing/apartment*  **Structure:**  12 months financial housing support for men. 66-person capacity.  **Support:** CM; referral to community-based services; peer-to-peer support.  **Restrictions:** Daily reporting. | 1. Education 2. Vocational skills & employment ^g^ 3. AOD use ^g^ 4. Mental health and wellbeing including anger management ^g^ 5. Physical health ^g^ 6. Life skills ^g^ | Not reported | Not reported |
| **Schwarz, 2020 (USA)** | Ethnographic case study | *1* | *Fixed-site*  **Structure:** 12-week housing for men.  **Support:** 24-hour staff availability; CM; on-site training  **Restrictions:** Sign-in and sign-out required by residents; 11pm curfew, Sunday-Friday. | 1. Education 2. Vocational skills & employment 3. AOD use 4. Mental health and wellbeing including anger management 5. Life skills 6. Financial skills | Stigma; self-stigma; self-esteem  (Participant-observation & self-report) | ↓ Stigma |
| **Nice, 1964 (USA)** | Descriptive | *1* | *Fixed-site*  **Structure:** Maximum 4 months accommodation for men. 21-person capacity. Employed residents are charged for room and board ($2 for every $10 earned).  **Support:** Group therapy.  **Restrictions:** Not reported | 1. Permanent housing 2. Vocational skills & employment 3. Financial skills 4. Connections to family & community | Not reported. | Not reported |

Notes: ^a^PCS=Prospective cohort study, CBA=Controlled before-and-after study, CSS=Cross-sectional study, RCS=Retrospective cohort study, QPE=Qualitative program evaluation, BAC=Before-and-after comparison, MM=Mixed methods, MCS=Multiple case study.

^b^ Lutze, 2009 and Lutze, 2014 refer to three services, with Lutze, 2014 providing outcome and effectiveness details for the three services collectively, and Lutze, 2009 providing detailed information about the targeted factors and description of each service.

^c^AD=Administrative data

^d^IR=Incidence rate

^e^Indicates reporting on impact of specific program components on client outcomes.

^f^ Indicates comparison of all program participants (including those who did not complete the intervention) with control group. Comparison between completers and non-completers (rather than matched comparison participants) may have produced different findings.

^g^ Services provided by external services.

## *Indicates a statistically significant result, if a statistical comparison is reported
