# Supplementary material for: The impact of supported accommodation on health and criminal justice outcomes of people released from prison: a systematic literature review
Source: Harm Reduct J. 2023 Jul 21;20:91. doi: 10.1186/s12954-023-00832-8 (PMC10362610; doi:10.1186/s12954-023-00832-8)
Supplement: Supplementary file 2 — Additional file 2. Supported accommodation: A systematic review: Appendices. [file 12954_2023_832_MOESM2_ESM.docx]

## Appendix A. Search terms employed in electronic databases

### Table A. 1 Scopus search strategy

| **Word or phrase** | **#** | **Search term** | **Search date** | **Results** | **Search date** | **Results** | **Search date** | **Results** |
| --- | --- | --- | --- | --- | --- | --- | --- | --- |
| **People in or leaving custody** | 1 | prison* OR jail OR gaol OR incarcerat* OR felon OR offender OR recidivism OR re-offend* OR reoffend* OR "community corrections" OR imprison* OR ex-prisoner* OR ex-convict* OR ex-offender* | 5/9/20 | 127254 | 22/9/21 | 13,184 | 9/11/22 | 146,091 |
| **Release from custody** | 2 | post-release OR release* OR re-integrat* OR re-entry OR reintegrat* OR "leaving custody" OR "leaving prison" OR "leaving jail" OR "previously incarcerated" OR parole* OR probation | 5/9/20 | 1480794 | 22/9/21 | 140,622 | 9/11/22 | 1,674,023 |
| **Supported housing** | 3 | "supported accommodation" OR accommodation OR hous* OR homeless* OR living OR resettlement | 5/9/20 | 1509325 | 22/9/21 | 187,904 | 9/11/22 | 1,750,245 |
|  |  | 1 & 2 & 3 | 5/9/20 | 1321 | 22/9/21 | 220 | 9/11/22 | 1576 |

### Table A. 2 Medline search strategy

| **Word or phrase** | **#** | **Search term** | **Search date** | **Results** | **Search date** | **Results** | **Search date** | **Results** |
| --- | --- | --- | --- | --- | --- | --- | --- | --- |
| **People in or leaving custody** | 1 | prison* OR jail OR gaol OR incarcerat* OR felon OR offender OR recidivism OR re-offend* OR reoffend* OR "community corrections" OR imprison* OR ex-prisoner* OR ex-convict* OR ex-offender* | 5/9/20 | 38589 | 22/9/21 | 3,890 | 9/11/22 | 64,830 |
| **Release from custody** | 2 | post-release OR release* OR re-integrat* OR re-entry OR reintegrat* OR "leaving custody" OR "leaving prison" OR "leaving jail" OR "previously incarcerated" OR parole* OR probation | 5/9/20 | 659881 | 22/9/21 | 46,681 | 9/11/22 | 834,173 |
| **Supported housing** | 3 | "supported accommodation" OR accommodation OR hous* OR homeless* OR living OR resettlement | 5/9/20 | 554324 | 22/9/21 | 103,580 | 9/11/22 | 1,224,660 |
|  |  | 1 & 2 & 3 | 5/9/20 | 410 | 22/9/21 | 89 | 9/11/22 | 844 |

### Table A. 3 Embase search strategy

| **Word or phrase** | **#** | **Search term** | **Search date** | **Results** | **Search date** | **Results** | **Search date** | **Results** |
| --- | --- | --- | --- | --- | --- | --- | --- | --- |
| **People in or leaving custody** | 1 | prison* OR jail OR gaol OR incarcerat* OR felon OR offender OR recidivism OR re-offend* OR reoffend* OR "community corrections" OR imprison* OR ex-prisoner* OR ex-convict* OR ex-offender* | 5/9/20 | 67459 | 22/9/21 | 6680 | 9/11/22 | 75,549 |
| **Release from custody** | 2 | post-release OR release* OR re-integrat* OR re-entry OR reintegrat* OR "leaving custody" OR "leaving prison" OR "leaving jail" OR "previously incarcerated" OR parole* OR probation | 5/9/20 | 1153534 | 22/9/21 | 95553 | 9/11/22 | 1,278,071 |
| **Supported housing** | 3 | "supported accommodation" OR accommodation OR hous* OR homeless* OR living OR resettlement | 5/9/20 | 804426 | 22/9/21 | 105302 | 9/11/22 | 951,487 |
|  |  | 1 & 2 & 3 | 5/9/20 | 668 | 22/9/21 | 126 | 9/11/22 | 823 |

### Table A. 4 PsycInfo search strategy

| **Word or phrase** | **#** | **Search term** | **Search date** | **Results** | **Search date** | **Results** | **Search date** | **Results** |
| --- | --- | --- | --- | --- | --- | --- | --- | --- |
| **People in or leaving custody** | 1 | prison* OR jail OR gaol OR incarcerat* OR felon OR offender OR recidivism OR re-offend* OR reoffend* OR "community corrections" OR imprison* OR ex-prisoner* OR ex-convict* OR ex-offender* | 5/9/20 | 55127 | 22/9/21 | 2,064 | 9/11/22 | 83,248 |
| **Release from custody** | 2 | post-release OR release* OR re-integrat* OR re-entry OR reintegrat* OR "leaving custody" OR "leaving prison" OR "leaving jail" OR "previously incarcerated" OR parole* OR probation | 5/9/20 | 62114 | 22/9/21 | 1,844 | 9/11/22 | 68,794 |
| **Supported housing** | 3 | "supported accommodation" OR accommodation OR hous* OR homeless* OR living OR resettlement | 5/9/20 | 215820 | 22/9/21 | 10,185 | 9/11/22 | 304,565 |
|  |  | 1 & 2 & 3 | 5/9/20 | 888 | 22/9/21 | 53 | 9/11/22 | 1328 |

### Table A. 5 Social Sciences Citation Index search strategy

| **Word or phrase** | **#** | **Search term** | **Search date** | **Results** | **Search date** | **Results** | **Search date** | **Results** |
| --- | --- | --- | --- | --- | --- | --- | --- | --- |
| **People in or leaving custody** | 1 | prison* OR jail OR gaol OR incarcerat* OR felon OR offender OR recidivism OR re-offend* OR reoffend* OR "community corrections" OR imprison* OR ex-prisoner* OR ex-convict* OR ex-offender* | 5/9/20 | 111966 | 22/9/21 | 8,135 | 9/11/22 | 122,713 |
| **Release from custody** | 2 | post-release OR release* OR re-integrat* OR re-entry OR reintegrat* OR "leaving custody" OR "leaving prison" OR "leaving jail" OR "previously incarcerated" OR parole* OR probation | 5/9/20 | 1170773 | 22/9/21 | 81,419 | 9/11/22 | 1,334,163 |
| **Supported housing** | 3 | "supported accommodation" OR accommodation OR hous* OR homeless* OR living OR resettlement | 5/9/20 | 2389978 | 22/9/21 | 200,126 | 9/11/22 | 2,758,497 |
|  |  | 1 & 2 & 3 | 5/9/20 | 1661 | 22/9/21 | 196 | 9/11/22 | 2051 |

## Appendix B. Studies excluded at full text review stage and reasons for exclusion

| **Study** | **Title** | **Notes** |
| --- | --- | --- |
| **Fontaine 2013** | The Role of Supportive Housing in Successful Reentry Outcomes for Disabled Prisoners | Other - service is not transitional |
| **Terrell 1998** | A means for re-integrating African Americans convicted of non-violent crimes | Wrong publication type – Proposal, not a description or evaluation of an existing service |
| **Maier 2019** | Half way to freedom: The role of halfway houses in Canada's penal landscape. | Supported accommodation not sufficiently described |
| **Kennedy 1964** | Halfway Houses Pay Off | Paediatric population |
| **Hyatt 2018** | Expanding the focus of correctional evaluations beyond recidivism: the impact of halfway houses on public safety | Not post-release |
| **Growns 2018** | A Systematic Review of Supported Accommodation Programs for People Released From Custody | Wrong publication type – Systematic review |
| **Berry 2018** | Coming home: Challenges and opportunities to enhance reentry success. | Wrong publication type – Book chapter |
| **Jasni 2019** | Function Limitations of Halfway Houses as Temporary Homes Intended for Former Prisoners in Malaysia | Wrong publication type – Concept paper |
| **Maier 2020** | Canada's ‘Open Prisons’: Hybridisation and the Role of Halfway Houses in Penal Scholarship and Practice | Supported accommodation not sufficiently described |
| **Latessa 1982** | Halfway houses and parole: A national assessment | Supported accommodation not sufficiently described |
| **Seiter 2003** | Prisoner reentry: What works, what does not, and what is promising | Supported accommodation not sufficiently described |
| **Rojiani 2021** | Group drumming for incarcerated men may improve community reintegration: a mixed methods pilot study | Supported accommodation not sufficiently described |
| **Wesely 2021** | The Body, The Self: How Women Ex-Offenders in a Re-Entry Program Negotiate Gendered, Embodied Identities, and the Implications for Desistance | Supported accommodation not sufficiently described |
| **Soto-Nevarez 2021** | Housing, Relationships, and Substance Use among Formerly Incarcerated Females | Supported accommodation not sufficiently described |
| **Smoyer 2021** | Ping-Pong Housing: Women’s Post-Incarceration Trajectories | Supported accommodation not sufficiently described |
| **Maier 2020** | Intermediary workers: Narratives of supervision and support work within the halfway house setting | Supported accommodation not sufficiently described |
| **Jacobs 2020** | The Effect of Housing Circumstances on Recidivism: Evidence From a Sample of People on Probation in San Francisco | Supported accommodation not sufficiently described |
| **Rosenberg 2021** | “I don't know what home feels like anymore”: Residential spaces and the absence of ontological security for people returning from incarceration | Supported accommodation not sufficiently described |
| **Dubois 2020** | Challenges to reintegration : Barriers to reentry encountered by ex-convicts in halfway houses | Supported accommodation not sufficiently described |
| **Cram 2020** | The ‘carrot’ and ‘stick’ of integrated offender management: implications for police culture | Supported accommodation not sufficiently described |
| **Borowski 2020** | An Evaluation of the Community Recovery Program: A Case Management Approach to Assisting Individuals Recover from Substance Use and Incarceration | Supported accommodation not sufficiently described |
| **Blomberg 2020** | Digital divide and marginalized women during COVID-19: a study of women recently released from prison | Supported accommodation not sufficiently described |
| **Wong 2019** | Halfway Out: An Examination of the Effects of Halfway Houses on Criminal Recidivism | Wrong publication type – Systematic review |
| **Polcin 2018** | Role of recovery residences in criminal justice reform | Exclusive focus service (mental health, AOD, etc) |
| **Polcin 2006** | What about Sober Living Houses for Parolees? | Exclusive focus service (mental health, AOD, etc) |
| **Nyamathi 2016** | A randomized clinical trial of tailored interventions for health promotion and recidivism reduction among homeless parolees: outcomes and cost analysis | Exclusive focus service (mental health, AOD, etc) |
| **Barak 2021** | Anticipated Alienation and Critical Social Work: Ex-Offenders' Perspectives on Re-entry | Supported accommodation not sufficiently described |
| **Barkley 2000** | Back To The Future: Housing And Support For Offenders | Supported accommodation not sufficiently described |
| **Latessa 1982** | Halfway houses and parole: A national assessment. | Duplicate |
| **Kras 2016** | A New Way of Doing Time on the Outside: Sex Offenders' Pathways In and Out of a Transitional Housing Facility | Supported accommodation not sufficiently described |
| **Hennen 2013** | From handcuffs to home: Examining previously incarcerated mothers' experience living in a reentry transitional housing program: A phenomenological study. | Supported accommodation not sufficiently described |
| **McNeeley 2018** | Do ecological effects on recidivism vary by gender, race, or housing type? | Supported accommodation not sufficiently described |
| **Boone 2018** | Prisoner resettlement in the Netherlands: Great initiatives for too few people | Unable to locate full text |
| **Burnett 2010** | Post-corrections reintegration: Prisoner resettlement and desistance from crime | Unable to locate full text |
| **Engle 1999** | The rocky road home: making the transition from prison to the community. | Unable to locate full text |
| **Harford 2019** | Helping and hindering factors for inmates meeting the challenges for successful community reentry. | Not post-release |
| **Grimshaw 2004** | Prisoner resettlement and accommodation: Challenges for the new corrections | Supported accommodation not sufficiently described |
| **Day 1988** | Area Accommodation Strategies: A Partnership Approach to Housing for Homeless Offenders | Supported accommodation not sufficiently described |
| **Lantz 2018** | Using pay-for-success financing for supportive housing interventions: Promise & challenges | Non-carceral service |
| **Ducksworth 2010** | The prisoner reentry industry | Supported accommodation not sufficiently described |
| **Beausoleil 2017** | The effect and expense of redemption reintegration services versus usual reintegration care for young African Canadians discharged from incarceration | Supported accommodation not sufficiently described |
| **Sowers 2020** | Predictive factors of offender nonrecidivism through corrections department-funded transitional housing: An action research study | Supported accommodation not sufficiently described |
| **Martin 2018** | “Free but Still Walking the Yard”: Prisonization and the Problems of Reentry | Not post-release |
| **Paat 2017** | Hispanic exconvicts' perceptions of challenges and reintegration | Supported accommodation not sufficiently described |
| **Mellow 2008** | Transitioning Offenders to the Community: A Content Analysis of Reentry Guides | Supported accommodation not sufficiently described |
| **GOEKE 1993** | PERSONALITY-PATTERNS OF MALE FELONS IN A CORRECTIONAL HALFWAY HOUSE SETTING - AN MMPI TYPOLOGICAL ANALYSIS | Supported accommodation not sufficiently described |
| **Vasoli 1970** | Halfway House for Reformatory Releasees | Paediatric population |
| **Callahan 2016** | Reducing Economic Disparities for Female Offenders: The Oxford House Model | Exclusive focus service (mental health, AOD, etc) |
| **Day 2011** | Reintegration Services for Long-Term Dangerous Offenders: A Case Study and Discussion | Duplicate |
| **Crow 1980** | Working with the Discharged Prisoner | Unable to locate full text |
| **Garland 2013** | Value Conflict and Public Opinion Toward Prisoner Reentry Initiatives | Not post-release |
| **HARRIS 1994** | CLIENT MANAGEMENT CLASSIFICATION AND PREDICTION OF PROBATION OUTCOME | Not post-release |
| **Steiner 2015** | Examining the Effects of Residential Situations and Residential Mobility on Offender Recidivism | Supported accommodation not sufficiently described |
| **Cooper 2014** | Resettlement provision for adult offenders | Supported accommodation not sufficiently described |
| **Francois 2018** | Prison sociability and reintegration | Supported accommodation not sufficiently described |
| **Lewis 2007** | What works in resettlement?: Findings from seven Pathfinders for short-term prisoners in England and Wales. | Supported accommodation not sufficiently described |
| **Parsell 2017** | Cost Offsets of Supportive Housing: Evidence for Social Work | Non-carceral service |
| **Molyneux 2005** | A better future for supported housing | Non-carceral service |
| **Porter 1977** | A Framework for Comparison of Intensive and Special Probation Projects | Not post-release |
| **Mccall 1998** | Meeting the offender re-integration challenge | Supported accommodation not sufficiently described |
| **Latessa 1991** | Halfway house or probation: A comparison of alternative dispositions | Supported accommodation not sufficiently described |
| **Liem 2018** | Reintegration Among High-Profile Ex-Offenders | Supported accommodation not sufficiently described |
| **Salem 2015** | Supportive Housing and Forensic Patient Outcomes | Exclusive focus service (mental health, AOD, etc) |
| **Cherner 2014** | Transitioning into the Community: Outcomes of a Pilot Housing Program for Forensic Patients | Exclusive focus service (mental health, AOD, etc) |
| **Abrams 2008** | Transition services for incarcerated youth: A mixed methods evaluation study | Paediatric population |
| **Cluley 2009** | Resettlement factors and reducing re-offending | Supported accommodation not sufficiently described |
| **Schlitt 1980** | Developing prediction equations to increase success at three adult male offender halfway houses. | Unable to locate full text |
| **Garland 2017** | What Influences Public Support of Transitional Housing Facilities for Offenders During Reentry? | Supported accommodation not sufficiently described |
| **Maier 2020** | `Mobilizing' prisoner reentry research: Halfway houses and the spatial-temporal dynamics of prison release | Supported accommodation not sufficiently described |
| **SETTLE 1965** | EMERGENCY ACCOMMODATION FOR BOYS LEAVING PRISON | Paediatric population |
| **Lattimore 2014** | The impact of prison reentry services on short-term outcomes: Evidence from a multisite evaluation | Not post-release |
| **Martinez 2009** | The familial relationships of former prisoners: Examining the link between residence and informal support mechanisms. | Supported accommodation not sufficiently described |
| **Harding 2000** | Supporting People - Opportunities and Threats Facing the Housing and Support Needs of Offenders | Supported accommodation not sufficiently described |
| **Backman 2019** | Exploring halfway house mentors' lived experiences working with female offenders. | Supported accommodation not sufficiently described |
| **Dalkin 2004** | The reducing re-offending action plan and prisoner resettlement | Wrong publication type – Discussion of government report |
| **1991** | Ex-Offender Hostels : All Change | Supported accommodation not sufficiently described |
| **Cherner 2013** | Findings of a Formative Evaluation of a Transitional Housing Program for Forensic Patients Discharged into the Community | Exclusive focus service (mental health, AOD, etc) |
| **Zgoba 2020** | Assessing the Impact of Restrictive Housing on Inmate Post-Release Criminal Behavior | Not post-release |
| **Luther 2011** | An exploration of community reentry needs and services for prisoners: A focus on care to limit return to high-risk behavior | Supported accommodation not sufficiently described |
| **Jason 2015** | Evaluating Alternative Aftercare Models for Ex-Offenders | Exclusive focus service (mental health, AOD, etc) |
| **Perry 2008** | Reducing Crime: The Effectiveness of Criminal Justice Interventions | Not post-release |
| **Growns 2018** | A Systematic Review of Supported Accommodation Programs for People Released From Custody | Duplicate |
| **Lattimore 2013** | The impact of prison reentry services on short-term outcomes: Evidence from a multisite evaluation. | Duplicate |
| **Mallenhoff 2009** | Criminal reform: Prisoner reentry into the community | Unable to locate full text |
| **Nice 1966** | After-care treatment of the released offender. | Unable to locate full text |
| **Caputo 2014** | A halfway house for women: Oppression and resistance | Unable to locate full text |
| **Miller 2015** | A promising jail reentry program revisited: Results from a quasi-experimental design. | Duplicate |
| **Lutze 2014** | Homelessness and reentry: A multisite outcome evaluation of Washington State's reentry housing program for high risk offenders. | Duplicate |
| **Lattimore 2013** | The Impact of Prison Reentry Services on Short-Term Outcomes: Evidence From a Multisite Evaluation | Duplicate |
| **NICE 1964** | HALFWAY HOUSE AFTERCARE FOR THE RELEASED OFFENDER | Duplicate |
| **Routh 2015** | Work release as a transition: Positioning success via the halfway house. | Duplicate |
| **Tarpey 2016** | A place to call home: Perspectives on offender community reintegration. | Duplicate |
| **Riede 1976** | Symbolic modeling in preparation for halfway house placement. | Unable to locate full text |
|  | Discharge planning: reintegrating inmates living with HIV/AIDS into the community. | Unable to locate full text |
| **Vigessa 2016** | Who participates in reentry programming? An examination of women offenders in a midwestern state | Duplicate |
| **Hulmáková**  **2018** | Prisoner resettlement in the Czech Republic | Unable to locate full text |
| **Miller 2015** | A promising jail reentry program revisited: results from a quasi-experimental design | Not post-release |
| **Hartman 1994** | RESIDENTIAL PROBATION - A 7-YEAR FOLLOW-UP-STUDY OF HALFWAY HOUSE DISCHARGES | Duplicate |
| **Hamilton 2014** | Uncommonly observed: The impact of New Jersey's halfway house system. | Duplicate |
| **Bowman, 2020** | Voices of returning citizens: A qualitative study of a supportive housing program for ex-offenders in a rural community | Supported accommodation not sufficiently described |
| **Hobson, 2021** | Restorative Practices in Institutional Settings: The Challenges of Contractualised Support within the Managed Community of Supported Housing | Non-carceral service |
| **Martin, 2021** | Halfway house: Prisoner reentry and the shadow of carceral care | Wrong publication type - Book |
| **Ojeda, 2021** | Overview of a Pilot Health-focused Reentry Program for Racial/Ethnic Minority Probationers ages 18 to 26 in Southern California | No accommodation component |
| **Petersen, 1991** | Correlates of success in a correctional halfway house for first-time male felons | Unable to locate full text |

## Appendix C. Synthesis Without Meta-Analysis (SWiM) in Systematic Reviews

| **SWiM guideline criteria** | **Methods used in this review** |
| --- | --- |
| 1. Rationale provided for grouping studies in the synthesis | For aim 1 (describing characteristics) studies were grouped based on service structure, support provided and program components in order to identify common program components. For aim 2 (assessing impact) studies were grouped based on outcome type (criminal justice outcomes, housing outcomes and other health and mental wellbeing outcomes) to identify best evidence. |
| 2. Standardised metric chosen to assess outcome | Where data were available, we used direction of effect as the standardised metric to assess outcomes, as it allowed synthesis of diverse effect measures. |
| 3. Synthesis methods used | As per criterion 2, we synthesised data narratively based on vote counting of direction of effect. Where sufficient data were available, we generated direction of effect graphs to visually depict effect direction across the outcomes, incorporating ROB to facilitate interpretation. |
| 4. Criteria were used to prioritise results for summary and synthesis | To describe program characteristics, results were prioritised based on data availability. To assess impact of supported accommodation on client outcomes, results were prioritised based on study design, whereby studies with any form of comparison group were synthesised first. |
| 5. Investigation of heterogeneity in reported effects | Heterogeneity was assessed by examining outcomes based on study design and outcome measure. |
| 6. Certainty of evidence | We used applicable items from the GRADE framework (Schünemann et al., 2019), including risk of bias (McGill Mixed Methods Appraisal Tool (MMAT), indirectness, and publication bias. |
| 7. The method to present data | Data for each study were summarised in tabulated format, with certain outcomes presented graphically using the direction of effect plots (Boon & Thomson, 2021). |
| 8. Reporting results | We made clear which studies contributed to each synthesis via stating the total number in text, citing the study, and briefly describing the study design. |
| 9. Limitations of synthesis | We identified limitations of the synthesis and how these affect the conclusions that can be drawn. Limitations are described in the body of the manuscript. |

## Appendix D. Study details

| **Author & year of publication (country)** | **Study design**  ***Evaluation yes/no*** | **Aims of the paper** | **Program inclusion & exclusion criteria** | **Age range or mean age** | **(n) Sex/ gender** |
| --- | --- | --- | --- | --- | --- |
| **Simonds, 2022 (USA)** | Randomised control trial | Evaluate the impact of an RCT where formerly incarcerated individuals were provided with paid transitional housing before and during a COVID-19 stay-at-home order. | *Inclusion*  Began time at re-entry centre between September 2019 and March 2020.  *Exclusion*  Refusal to participate; indication of plans to live with family friends, or others after release from prison. | Mean 40 years | 315 men |
| **Willison, 2010 (USA)** | Controlled before and after  *Yes* | Measure the impact of Ridge House on participant recidivism compared to waitlist group | *Inclusion*  Interest in participating in the program  *Exclusion*  Sexual offence > tier one. | Treatment group: Mean: 36.6 years  Control:  Mean: 36.6 years | Treatment group: 156 clients; 69% men (n=108)  Control: 461; 75% men (n=356) |
| **Dowell, 1985 (USA)** | Controlled before and after  *Yes* | Identify whether there is evidence that the halfway house helps reduce recidivism or the severity of crimes committed | *Inclusion*  Committed at least one federal offense  *Exclusion*  Not described | Mean: 31.5 years | 60 women |
| **Routh, 2015 (USA)** | Prospective cohort study  *Yes* | 1. To evaluate the effectiveness of work release HWHs provided by the New Jersey Department of Corrections (NJDOC) 2. To examine potential effects of offender characteristics on the successful provision of HWH work release programs. | *Inclusion*  Those whose risk/needs assessment (conducted by the NJDOC) indicated a need for work release and met a certain risk level are deemed eligible.  *Exclusion*  Not detailed. | Mean: 34 years | 25,459 |
| **Hamilton, 2014 (USA)** | Prospective cohort study  *No* | Compare recidivism rates between those who access a halfway house in New Jersey with those who don’t participate. | *Inclusion*  Within 24 months of parole eligibility and/or completion of sentence, and approved for community release programs. LSI-R  *Exclusion*  Not described | Treatment group: mean 34.9 years  Comparator: mean 34.8 years | Treatment group: 88% men (n=5807)  Comparator: 88.5% men (n=5840) |
| **Lutze, 2014 (USA)** | Prospective cohort study  *Yes* | Outcome evaluation of the Washington State Reentry Housing Pilot Program, comparing clients of the services with those under traditional supervision | *Inclusion*  High risk/need inmates without a viable release plan with 12 months community supervision, currently incarcerated for their initial sentence, eligible for release January 2008-July 2009, volunteered to participate  *Exclusion*  Major infractions within 90 days, warrant or detainer | Mean 39.4 years | Treatment group: 21.2% women (n=44)  Comparison group: 18% women. (n=37) |
| **Lowenkamp, 2002 (USA)** | Prospective cohort study  *Yes* | Describe Ohio’s Halfway House and Community Based Corrections Facility programs and evaluate the outcomes of each program and each grouping of programs (HWH and CBCF). | *Inclusion*  Not detailed.  *Exclusion*  Not detailed. | Mean age: 33.5 | 3,737 (experimental group) and 3,058 (comparison group)  Men and women |
| **Shoham, 2021 (Israel)** | Retrospective cohort study  *Yes* | Investigate rates of recidivism among prisoners on parole with a substance misuse disorder who participate in the Jerusalem halfway-house | *Inclusion*  Halfway house intended for those released from prison with characteristics defined as difficult or complex (many years AOD use, high recidivism, and lacking family or social sources of support).  *Exclusion*  Not detailed | Treatment group completers mean: 33.1 years  Matched control: 34 | Treatment group completers: 40 men  Matched control: 40 men |
| **Davies, 2018 (UK)** | Cross sectional study  *No* | Establish a baseline of resident and staff experience, attitudes, and well-being as well as formally recorded resident outcomes | *Inclusion*  Men who have committed serious violent or sexual offending, who are categorised as high or very high risk of serious harm to themselves or others  *Exclusion*  Not described | Average: 42 years | 114 men |
| **Hignite, 2017 (USA)** | Cross sectional study  *No* | Assess the initial programmatic success of a post-incarceration support service in Houston, Texas | *Inclusion*  Homelessness; annual income <50% of area median income; US citizenship or valid ID.  *Exclusion*  No described | Not reported | 76 clients; 75% men (n=57) |
| **Vigessa, 2016 (USA)** | Cross sectional study    *No* | Examine the relationship between demographic,  social, and criminal history variables and participation  in one of three prison release mechanisms: (a) direct release to parole, (b) reentry programming (provision of various treatment and halfway house accommodation while still inmates), and (c) halfway house (for parolees). | *Inclusion*  Criteria related to risk levels, conviction and incarceration  History mentioned but not detailed.  *Exclusion*  Not detailed. | Mean age: 30.82 | 295 women |
| **Morley, 2014 (USA)** | Cross sectional study  *No* | Evaluate the relationship between individual characteristics and length of time in a treatment program | *Inclusion*  Current or anticipated parole from an IDOC adult facility  *Exclusion*  History of sexual offences or offences against children.  Juvenile Justice involvement  Complex physical or mental health needs requiring supportive infrastructure  Inpatient substance abuse detoxification | Mean 41 years (SD 11.4) | 103 men |
| **Ellison, 2013 (UK)** | Cross sectional study  *Yes* | Evaluate the impact of Vision Housing’s provision of housing and support on re-offending rates  Seeks to evaluate the impact of Vision Housing’s  provision of housing and support on re-offending rates. | *Inclusion*  No specific inclusion criteria. Typical client are persistent, prolific adult offenders.  *Exclusion*  Convicted of an arson offence or violent sexual offence. | Range: 15-64 years | 400 clients; 82.5% men (n=329), 17.5% women (n=70)  Vision Housing has supported over 650 clients. |
| **Yeboah, 2000 (New Zealand)** | Cross sectional study  *No* | Summary of key findings of evaluation of the Habilitation Centre’s Pilot Programme (Salisbury Street Foundation [SSF] and Aspell House [AH]) | *Inclusion*  Serving sentence of ≥12 months. Consent to conditions of release orders  *Exclusion*  On corrective training | Range: 16-60 years (AH)  Range 21-43 years (SSF) | 30 women (AH)  39 men (SFS) |
| **Hartman, 1994 (USA)** | Cross sectional study  *No* | Analyse predictors of successful program discharge and recidivism over a seven-year period following discharge of 156 subjects from a probation halfway house | *Inclusion*  Conviction of a non-violent crime  *Exclusion*  Not described | Mean 19.8 years (range 17-32) | 156 clients; 96.2% men (n=150) |
| **Calathes, 1991 (USA)** | Cross sectional study  *No* | Describe Project Green Hope and report on characteristics of clients relating to program success or failure. | *Inclusion*  Not described  *Exclusion*  Not described | “Young” (age not reported) | Women (number not specified) |
| **Walsh, 1990 (USA)** | Cross sectional study    *No* | To investigate factors that  may have an impact on recidivism among halfway house residents. | *Inclusion*  Not detailed.  *Exclusion*  Not detailed. | Age range: 19-49  Mean age: 28.3 (SD=6.7) | 75 men |
| **Donnelly, 1984 (USA)** | Cross sectional study  *No* | Describe a supported accommodation services program and examine the diversity of clients entering the program over a 3-year period | *Inclusion*  Not reported  *Exclusion*  Prospective residents who are not accepted normally include chronic violent offenders, as well as being charged with sex crimes or arson, people with complex AOD needs, or complex mental health problems. | Median: 26.6 years | 409 clients, 68% men (n=276) |
| **Twill, 1998 (USA)** | Before and after  *No* | Examine loneliness, social support, and locus of control as related to the successful readjustment of parolees living in a halfway house | *Inclusion*  Participation in program as condition of release  *Exclusion*  Not described | Mean 33 years (SD 7.2) | 37 men |
| **Techagaisiyavanit, 2021 (Thailand)** | Mixed methods study  *No* | To examine the experiences of and attitudes towards the Kalatapae halfway house from the perspectives of HWH residents, HWH staff, local government officials and local community residents. | Not detailed. | Age range: 22-43  Mean age: 30.6 | 22 men |
| **Williams, 2003 (USA)** | Mixed methods study  *No* | To explore quality of life perceptions of offenders and subsequent therapeutic implications | *Inclusion*  Probationers ordered to complete sex offender treatment by the courts  Parolees referred by the prison system  *Exclusion*  Civil commitments or people after sentence expiration | Mean 31 years (SD 12.24) | 23 men |
| **Pleggenkuhle, 2016**  **(USA)** | Qualitative program evaluation    *No* | Compare the reentry needs among a sample of male parolees participating in a housing program (Solid Start) with a similar sample of males on traditional parole | *Inclusion*  1 or more of: Long incarceration (traditionally >10years); Little social support in community; Substantial child support or other financial obligations; No consistent work history; ‘Maxed out’ prison sentence; Mild-to-moderate mental health disorder  *Exclusion*  Unwilling to abide by program guidelines, or severe mental health illness or substance abuse needs | Treatment group: mean 40.8 years  Comparison group: mean 34.2 years | Treatment group: 100% men (n=18)  Comparison group: 100% men (n=18) |
| **Birgerson, 2022 (Australia)** | Multiple case study  *No* | Examine the consequences of removing a transitional accommodation support service on service providers and returning citizens. | *Inclusion*  Sentences longer than 6 months and at risk of homelessness post-release.  *Exclusion*  Not reported | Not reported | 16 people |
| **Tarpey, 2016**  **(UK)** | Multiple case study    *No* | Explore the experience of community reintegration from the perspective of UK housing scheme residents following release from incarceration | *Not described* | Range 25-55 years | 3 men  2 women |
| **Day, 2011 (Australia)** | Multiple case study    *No* | Describe the results of an external review of an offender reintegration program delivered by an NGO. | *Inclusion*  Long-term offenders who have been identified as dangerous offenders.  *Exclusion*  Not described | Average: 40 years | 50 men |
| **Lutze, 2009 (USA)** | Report  *No* | Describe findings from first year of Re-entry Housing Pilot Program operation (RHPP) | *Inclusion*  Minimum 12 months community supervision to serve; currently incarcerated on initial sentence; county of origin in a pilot setting of the RHPP; high risk, high need client, or without viable release plan.  *Exclusion*  Major infraction within last 90 days; felony warrant. | Mean: 38.4 years | 106 clients; 79% men (n=84) |
| **Schwarz, 2020 (USA)** | Ethnographic case study  *No* | To investigate whether or not a halfway house provides a metaphorical  “safe umbrella” from criminal stigmatization for previously  incarcerated individuals | *Inclusion*  Criminal background  *Exclusion*  History of sexual offences  *NB: Selection by intake worker includes “individual’s eagerness to live a lawful lifestyle”* | Not reported | 18 men |
| **Nice, 1964 (USA)** | Descriptive  *No* | To describe historical transitional housing attempts throughout Europe and more recent American housing programs, with a focus on Robert Bruce House (New Jersey). | *Inclusion*  Applicants (1) must appear to need the service, (2) must come from the geographical area surrounding the house, (3) must have no family or friends to whom they could be paroled, (4) must be participating in some form of therapy at the New Jersey State Reformatory (NJSR), and (5) must volunteer for the project.  *Exclusion*  The program is only available to parolees of the NJSR. | Not reported | 21 men |

## Appendix E. Program component definitions

| **Term** | **Study** | **Definitions/components described in literature** |
| --- | --- | --- |
| **Permanent housing** | Willison, 2010 | Stable, drug-free housing. |
|  | Pleggenkuhle, 2016 | Stepped down rental coverage after 3 months, full rent takeover by client at 12 months. |
|  | Calathes, 1991 | Initiate plan for permanent housing, with assistance from staff housing specialist, working with public and private networks to locate permanent housing. |
|  | Day, 2011 | Assistance provided to find permanent accommodation. Definition not provided. |
|  | Donnelly, 1984 | Making satisfactory efforts towards securing housing considered a core of program success/completion. |
|  | Tarpey, 2016 | Progression onto a standard tenancy within the housing association. |
|  | Nice, 1964 | Parole department approved residence in the community. |
|  | Lowenkamp, 2002 | Assistance with accommodation. |
|  | Lutze, 2009 | Housing assistance definition not provided. |
| **Education** | Lowenkamp, 2002 | Definition of education programs not provided |
|  | Yeboah, 2000 | Includes enrolment in formal courses. Further details not provided |
|  | Morley, 2016 | GED classes offered, as well as referral for other educational programming |
|  | Calathes, 1991 | Clients enrolled in educational or training program. Further details not provided |
|  | Day, 2011 | Clients assisted with educational or training program. Further details not provided |
|  | Walsh, 1990 | Attempt to provide an atmosphere as well as resources for additional education. No further details provided |
|  | Lutze, 2009 | Definition of education programs not provided |
|  | Hignite, 2017 | GED classes as well as referral to other educational programmes. |
|  | Schwarz, 2020 | High school diploma, financial literacy, computer, tutoring services |
|  | Hamilton, 2014 | Definition of education programs not provided |
|  | Birgerson, 2022 | Assisted with integral life skills, such as budgeting, education, and employment through an individual case plan. No further details provided. |
|  | Simonds, 2022 | Provide a wide range of services, such as employment, education, life skills training, substance-use treatment and counselling. No further details provided. |
| **Vocational skills & employment** | Willison, 2010 | Career development services. Employability training. |
|  |  | Clients are expected to obtain and maintain gainful employment after the first 30 days in the program. |
|  | Routh, 2015 | Work-release HWH programs. Further details not provided. |
|  | Lowenkamp, 2002 | Vocational training. No further details provided |
|  | Shoham, 2021 | Employment solutions and guidance. No further details provided |
|  | Hamilton, 2014 | Vocational training and employment services as a means to help subjects graduate from temporary employment to job placements within the community. |
|  | Hartman, 1994 | Employment skills classes meant for residents who are unemployed at admission, and which are designed to build job-seeking and retention skills; and job club, a peer support group for residents who are seeking or have recently obtained employment |
|  | Williams, 2003 | Clients are employed within the community. |
|  | Twill, 1998 | Required to search for employment six days a week until gainful employment is secured, and to participate in a job search group each day while unemployed, covering topics such as completing job applications and interviewing skills |
|  | Morley, 2016 | Vocational training and employment preparation available for all residents. Additionally, residents can participate in programs such as culinary arts training |
|  | Calathes, 1991 | Vocational rehabilitation counsellor providing assistance with vocational aptitude for different types of work, and evaluation of work readiness |
|  | Hignite, 2017 | Vocational programs not defined. |
|  | Techagaisiyavanit, 2021 | Career building programs with local industry |
|  | Day, 2011 | Assistance with employment. No further details provided |
|  | Donnelly, 1984 | Employment amongst needs addressed by service. No further details provided |
|  | Walsh, 1990 | Employment assistance. No further details provided |
|  | Lutze, 2009 | Definition of employment/vocational programs not provided |
|  | Tarpey, 2016 | Definition of employment/vocational programs not provided |
|  | Nice, 1964 | Group therapy addresses employment and employee-employer relationships |
|  | Schwarz, 2020 | Assistance with employment search, mock interviews and employment coaching; construction and culinary classes |
|  | Birgerson, 2022 | Assisted with integral life skills, such as budgeting, education, and employment through an individual case plan. No further details provided. |
|  | Simonds, 2022 | Provide a wide range of services, such as employment, education, life skills training, substance-use treatment and counselling. No further details provided. |
| **AOD use** | Willison, 2010 | AOD counselling (12-step). Substance abuse education. |
|  | Lowenkamp, 2002 | Counselling for alcohol and/or other drugs. Considerable variation in the types of programs offered. No further details provided. |
|  | Ellison, 2013 | Substance misuse services through third-party sector providers. |
|  | Shoham, 2021 | Treatment and rehabilitation programs designed for people who have experienced incarceration who have substance use disorders. Customized therapy, attending Narcotics Anonymous (NA) sessions, and frequent urine testing. |
|  | Hamilton, 2014 | Substance abuse treatment coupled with greater restrictions placed on community movements compared with traditional work release facilities. |
|  | Yeboah, 2000 | Community based Alcoholics Anonymous and Drugs Anonymous. |
|  | Hartman, 1994 | Substance abuse counselling offered through local agencies. |
|  | Morley, 2014 | Intensive Outpatient services. |
|  | Calathes, 1991 | Intensive substance abuse counselling. |
|  | Hignite, 2017 | Linkage to substance abuse prevention services. |
|  | Techagaisiyavanit, 2021 | Group therapy. Support group. Recovering addict[s] play a key role in providing guidance and leading activities. |
|  |  | Harm reduction principles toward reducing negative consequences of drug abuse. |
|  | Day, 2011 | Joint case management with AOD services. |
|  | Donnelly, 1984 | Community based Alcoholics Anonymous. |
|  | Lutze, 2009 | Referral to community-based substance abuse treatment. |
|  | Schwarz, 2020 | Substance abuse treatment services (relapse prevention, Alcoholic Anonymous, Narcotics Anonymous meetings). |
|  | Birgerson, 2022 | Try to increase participants’ self-esteem, instil a positive future outlook, decrease self-harm and the risk of suicide, decrease alcohol and drug dependency and reduce anxiety and negative side effects of incarceration. No further details provided. |
| **Mental health and wellbeing, including anger management** | Willison, 2010 | Mental health evaluation. |
|  | Lowenkamp, 2002 | Counselling for mental health, anger management counselling. No further details provided. |
|  | Ellison, 2013 | Referral to community-based mental health services. |
|  | Hamilton, 2014 | Responsive special needs treatment for mental health. No further details provided. |
|  | Morley, 2014 | Mandatory individual therapy and psychological assessment; group therapy including anger management group focused on identifying triggers, and cognitive behavioural therapy triad. |
|  | Hignite, 2017 | Referral to community-based mental health services. |
|  | Donnelly, 1984 | Referral to community based mental health services. |
|  | Walsh, 1990 | Referral to community-based mental health services. |
|  | Lutze, 2009 | Referral to community-based mental health services, as well as on-site treatment for one accommodation site. |
|  | Schwarz, 2020 | Mental health counselling (cognitive behavioural therapy, anger management, psychological assessment) |
|  | Yeboah, 2000 | Anger management activities. No further details provided. |
|  | Birgerson, 2022 | Try to increase participants’ self-esteem, instil a positive future outlook, decrease self-harm and the risk of suicide, decrease alcohol and drug dependency and reduce anxiety and negative side effects of incarceration. No further details provided. |
|  | Simonds, 2022 | Provide a wide range of services, such as employment, education, life skills training, substance-use treatment and counselling. No further details provided. |
| **Physical heath** | Ellison, 2013 | Clients often have problems with physical health, for which referrals to third-party services is provided. |
|  | Willison, 2010 | Physical health programs not defined. |
|  | Yeboah, 2000 | Recreational activities include swimming, kayaking, rafting and visits to the gym. |
|  | Walsh, 1990 | Assistance with physical health treatment. |
|  | Lutze, 2009 | Physical health programs not defined. |
| **Life skills** | Lutze, 2014 | Self-sufficiency plans, renter’s rights courses, and coordinated safety plans to address issues that may arise for landlords, neighbours, or the community related to high-risk offender behaviour. |
|  | Willison, 2010 | Provision of life-skill services. Definition of lifestyle skills not provided. |
|  | Pleggenkuhle, 2016 | Financial and life skills encouraged through gradual transfer of financial responsibilities to the client. |
|  | Lowenkamp, 2002 | Definition of life/parenting skills not provided. |
|  | Dowell, 1985 | Skills necessary to “go straight”. |
|  | Shoham, 2021 | Acquisition of life skills listed as one of the factors targeted to acquire the functional and emotional tools necessary for managing daily life. |
|  | Yeboah, 2000 | Definition of lifestyle skills not provided |
|  | Vigesaa, 2016 | Definition of life skills programs not provided. |
|  | Day, 2011 | Definition of life skills programs not provided. |
|  | Hartman, 1994 | Basic life skills classes emphasizing things like personal budgeting |
|  | Morley, 2014 | Residents participate in a life skills group that includes the following topics: health, household, money, recreation, safety, socialization skills, survival, time management, decision making and critical thinking, prioritizing, family skills, and coping skills. |
|  | Hignite, 2017 | Problem solving counselling. |
|  | Techagaisiyavanit, 2021 | Positive experiences and supportive reactions from the house and community play an important role in developing the life skills residents will need when facing future opportunities and challenges. |
|  | Lutze, 2009 | Definition of life skills programs not provided. |
|  | Tarpey, 2016 | Definition of life skills programs not provided. |
|  | Schwarz, 2020 | Financial literacy, computer skills, job placement and retention |
|  | Birgerson, 2022 | Assisted with integral life skills, such as budgeting, education, and employment through an individual case plan. No further details provided. |
|  | Simonds, 2022 | Provide a wide range of services, such as employment, education, life skills training, substance-use treatment and counselling. No further details provided. |
| **Financial skills** | Willison, 2010 | Financial management services provided. |
|  | Pleggenkuhle, 2016 | Financial and life skills encouraged through gradual transfer of financial responsibilities to the client. |
|  | Lowenkamp, 2002 | Financial management skills. No further details provided. |
|  | Ellison, 2013 | Clients often have problems with debt which are targeted through third-party sector providers. |
|  | Shoham, 2021 | Independent financial management tools. No further details provided. |
|  | Yeboah, 2000 | Budgeting activities. |
|  | Hartman, 1994 | Personal budgeting classes. |
|  | Vigessa, 2016 | Pay-to-stay program designed to help women gain control of their finances. |
|  | Calathes, 1991 | Vocational appointments which include focus on banking and budgeting skills. |
|  | Hignite, 2017 | Money management skills |
|  | Day, 2011 | Financial counselling |
|  | Donnelly, 1984 | Financial needs of clients addressed. |
|  | Walsh, 1990 | Financial counselling and financial resources provided. |
|  | Lutze, 2009 | Independent financial management tools. |
|  | Tarpey, 2016 | Budgeting programs not defined. |
|  | Nice, 1964 | Teaching budgeting a core objective. No further details provided. |
|  | Schwarz, 2020 | Financial literacy classes. |
|  | Birgerson, 2022 | Assisted with integral life skills, such as budgeting, education, and employment through an individual case plan. No further details provided. |
| **Self-esteem/self-efficacy** | Willison, 2010 | Identified as an important dimension of service provision by staff. |
|  | Pleggenkuhle, 2016 | Subjective factors, including self-concept identified as important influence on desistance. |
|  | Yeboah, 2000 | Modules on self-esteem. No further details provided. |
|  | Calathes, 1991 | Lack of self-esteem identified as a causative factor involved in women’s criminal justice-related problems. |
|  | Techagaisiyavanit, 2021 | Designing their own training programs has been used as a strategy to promote their personal self-esteem and responsibility in diverting themselves away from their old paths. |
|  | Birgerson, 2022 | Try to increase participants’ self-esteem, instil a positive future outlook. No further details provided. |
| **Spirituality and faith connections** | Willison, 2010 | Faith/spirituality services, including integration of faith/spirituality into other programs. |
|  | Lowenkamp, 2002 | Definition of spirituality services not provided. |
|  | Techagaisiyavanit, 2021 | Performance of religious practice part of daily routine. |
| **Connections to family and community** | Willison, 2010 | Relationship skills training. |
|  | Routh, 2015 | Clients are supported to resocialize to the community, to (re)learn social norms, prosocial behaviours attitudes and behaviours. |
|  | Shoham, 2021 | Family rehabilitation. Engagement in ‘cultural and enrichment events’ is encouraged. |
|  | Yeboah, 2000 | Program aims to contribute to the successful reintegration of offenders into the community and foster community involvement through the provision of programmes. |
|  | Twill, 1998 | Developing support groups in the community. |
|  | Morley, 2016 | Life skills classes include family skills components. |
|  | Techagaisiyavanit, 2021 | The feeling of belonging and being accepted by the community impact on a change in lifestyle for clients. |
|  | Day, 2011 | General support in establishing community networks and re-establishing family relationships. |
|  | Donnelly, 1984 | Programming emphasizes the development of social and psychological "roots" in the community as well as support groups. |
|  | Lutze, 2009 | Family rehabilitation. |
|  | Tarpey, 2016 | Scheme focus is reintegration into the community, aiming to promote lifestyle change |
|  | Nice, 1964 | The residents selected have no family, friends, or other community resources. |
| **First Nations cultural connections** | Yeboah, 2000 | Māori cultural connection programs. |
| **Specific criminal behaviour** | Williams, 2003 | Therapeutic intervention for people charged with sexual offense |
|  | Lowenkamp, 2002 | Sex offender treatment not defined. |
|  | Willison, 2010 | Criminal thinking counselling |
|  | Lutze, 2009 | Sex offender treatment services provided for one accommodation site. |
| **Domestic violence** | Lowenkamp, 2002 | Definition of domestic violence services not provided. |
|  | Willison, 2010 | Definition of domestic violence services not provided. |
|  | Hignite, 2017 | Referral to community-based domestic violence services. |
|  | Lutze, 2009 | Domestic violence programs not defined. |
| **Gender-specific** | Yeboah, 2000 | Women-specific accommodation site. |
|  | Lowenkamp, 2002 | Definition of women’s health programs not provided. |

## Appendix F. Mixed Methods Appraisal Tool criteria for specific study types

### Assessment of randomized controlled trials

Studies are assessed based on: 1. Appropriateness of randomization; 2. Comparability of groups at baseline; 3. Completeness of outcome data; 4. Blinding of outcome assessors to the intervention provided; and 5. Whether participants adhered to the assigned intervention.

### Assessment of quantitative non-randomized studies

Methodological quality criteria for quantitative non-randomized studies are: 1. Representativeness of participants; 2. Appropriateness of measurements regarding both the outcome and intervention groups; 3. Completeness of outcome data; 4. Accounting for confounders in design and analysis; and 5. Administration of intervention as intended throughout study period.

### Assessment of quantitative descriptive studies

Quantitative descriptive studies were appraised based on: 1. Relevance of sampling stratey to addressing the research question; 2. Representativeness of sample to target population; 3. Appropriateness of measurements; 4. Risk of non-response bias; and 5. Appropriateness of statistical analysis to the research question.

### Assessment of qualitative studies

The methodological quality of qualitative studies was assessed based on: 1. Appropriateness of the qualitative approach to the research question; 2. Whether the qualitative data collection methods were adequate to address the research question; 3. Whether the findings were adequately derived from the data; 4. Whether the interpretation of results was sufficiently substantiated by the data; and 5. Coherence between qualitative data sources, collection, analysis and interpretation.

### Assessment of mixed-methods studies

Mixed-methods studies were appraised based on: 1. Presence of an adequate rationale for using a mixed methods design; 2. Whether different components of the study are effectively integrated to answer the research question; 3. Whether outputs of the integration of qualitative and quantitative components are adequately interpreted; 4. Whether divergences and inconsistencies between quantitative and qualitative results are adequately addressed; and 5. Whether different components of the study adhere to the quality criteria of each tradition of the methods involved.

## Appendix G. Critique of methodological quality of studies using MMAT

| **Author & year of publication** | **Screening questions** | | **Qualitative studies** | | | | | **Quantitative randomized studies** | | | | | **Quantitative non-randomized studies** | | | | | **Quantitative descriptive studies** | | | | | **Mixed methods studies** | | | | |
| --- | --- | --- | --- | --- | --- | --- | --- | --- | --- | --- | --- | --- | --- | --- | --- | --- | --- | --- | --- | --- | --- | --- | --- | --- | --- | --- | --- |
|  | 1.1 | 1.2 | 2.1 | 2.2 | 2.3 | 2.4 | 2.5 | 3.1 | 3.2 | 3.3 | 3.4 | 3.5 | 4.1 | 4.2 | 4.3 | 4.4 | 4.5 | 5.1 | 5.2 | 5.3 | 5.4 | 5.5 | 6.1 | 6.2 | 6.3 | 6.4 | 6.5 |
| Lutze, 2014 | 🗸 | 🗸 |  |  |  |  |  |  |  |  |  |  | 🗸 | 🗸 | 🗸 | 🗸 | 🗸 |  |  |  |  |  |  |  |  |  |  |
| Shoham, 2021 | 🗸 | 🗸 |  |  |  |  |  |  |  |  |  |  | 🗸 | 🗸 | 🗸 | 🗸 | 🗸 |  |  |  |  |  |  |  |  |  |  |
| Willison, 2010 | 🗸 | 🗸 |  |  |  |  |  |  |  |  |  |  | 🗸 | 🗸 | 🗸 | 🗸 | 🗸 |  |  |  |  |  |  |  |  |  |  |
| Hartman, 1994 | 🗸 | 🗸 |  |  |  |  |  |  |  |  |  |  | 🗸 | 🗸 | 🗸 | 🗸 | 🗸 |  |  |  |  |  |  |  |  |  |  |
| Routh, 2015 | 🗸 | 🗸 |  |  |  |  |  |  |  |  |  |  | 🗸 | 🗸 | * | 🗸 | 🗸 |  |  |  |  |  |  |  |  |  |  |
| Ellison, 2013 | 🗸 | 🗸 |  |  |  |  |  |  |  |  |  |  | 🗸 | 🗸 | 🗸 | * | 🗸 |  |  |  |  |  |  |  |  |  |  |
| Davies, 2018 | 🗸 | 🗸 |  |  |  |  |  |  |  |  |  |  | 🗸 | 🗸 | 🗸 | * | 🗸 |  |  |  |  |  |  |  |  |  |  |
| Dowell, 1985 | 🗸 | 🗸 |  |  |  |  |  |  |  |  |  |  | 🗸 | 🗸 | 🗸 | 🗸 | X |  |  |  |  |  |  |  |  |  |  |
| Morley, 2016 | 🗸 | 🗸 |  |  |  |  |  |  |  |  |  |  | 🗸 | 🗸 | 🗸 | X | 🗸 |  |  |  |  |  |  |  |  |  |  |
| Vigessa, 2016 | 🗸 | 🗸 |  |  |  |  |  |  |  |  |  |  | 🗸 | 🗸 | 🗸 | * | * |  |  |  |  |  |  |  |  |  |  |
| Hamilton, 2014 | 🗸 | 🗸 |  |  |  |  |  |  |  |  |  |  | 🗸 | 🗸 | 🗸 | * | * |  |  |  |  |  |  |  |  |  |  |
| Hignite, 2017 | 🗸 | 🗸 |  |  |  |  |  |  |  |  |  |  | 🗸 | 🗸 | X | * | 🗸 |  |  |  |  |  |  |  |  |  |  |
| Calathes, 1991 | 🗸 | 🗸 |  |  |  |  |  |  |  |  |  |  | 🗸 | 🗸 | X | * | * |  |  |  |  |  |  |  |  |  |  |
| Tarpey, 2016 | 🗸 | 🗸 | 🗸 | 🗸 | 🗸 | 🗸 | 🗸 |  |  |  |  |  |  |  |  |  |  |  |  |  |  |  |  |  |  |  |  |
| Schwarz, 2020 | 🗸 | 🗸 | 🗸 | 🗸 | 🗸 | 🗸 | 🗸 |  |  |  |  |  |  |  |  |  |  |  |  |  |  |  |  |  |  |  |  |
| Pleggenkuhle, 2016 | 🗸 | 🗸 | 🗸 | 🗸 | 🗸 | 🗸 | 🗸 |  |  |  |  |  |  |  |  |  |  |  |  |  |  |  |  |  |  |  |  |
| Day, 2011 | 🗸 | 🗸 | 🗸 | 🗸 | 🗸 | 🗸 | 🗸 |  |  |  |  |  |  |  |  |  |  |  |  |  |  |  |  |  |  |  |  |
| Birgerson, 2022 | 🗸 | 🗸 | 🗸 | 🗸 | 🗸 | 🗸 | 🗸 |  |  |  |  |  |  |  |  |  |  |  |  |  |  |  |  |  |  |  |  |
| Donnelly, 1984 | 🗸 | 🗸 |  |  |  |  |  |  |  |  |  |  |  |  |  |  |  | 🗸 | 🗸 | 🗸 | 🗸 | 🗸 |  |  |  |  |  |
| Lutze, 2009 | 🗸 | 🗸 |  |  |  |  |  |  |  |  |  |  |  |  |  |  |  | 🗸 | 🗸 | 🗸 | 🗸 | 🗸 |  |  |  |  |  |
| Walsh, 1990 | 🗸 | 🗸 |  |  |  |  |  |  |  |  |  |  |  |  |  |  |  | 🗸 | 🗸 | 🗸 | 🗸 | * |  |  |  |  |  |
| Twill, 1998 | 🗸 | 🗸 |  |  |  |  |  |  |  |  |  |  |  |  |  |  |  | 🗸 | 🗸 | 🗸 | * | 🗸 |  |  |  |  |  |
| Calathes, 1991 | * | * |  |  |  |  |  |  |  |  |  |  |  |  |  |  |  | 🗸 | 🗸 | 🗸 | * | 🗸 |  |  |  |  |  |
| Williams, 2003 | 🗸 | 🗸 |  |  |  |  |  |  |  |  |  |  |  |  |  |  |  |  |  |  |  |  | 🗸 | 🗸 | 🗸 | 🗸 | 🗸 |
| Techagaisiyavanit, 2021 | 🗸 | 🗸 |  |  |  |  |  |  |  |  |  |  |  |  |  |  |  |  |  |  |  |  | 🗸 | 🗸 | 🗸 | * | 🗸 |
| Lowenkamp, 2002 | 🗸 | 🗸 |  |  |  |  |  |  |  |  |  |  |  |  |  |  |  |  |  |  |  |  | 🗸 | 🗸 | 🗸 | * | * |
| Yeboah, 2000 | 🗸 | 🗸 |  |  |  |  |  |  |  |  |  |  |  |  |  |  |  |  |  |  |  |  | 🗸 | 🗸 | 🗸 | * | * |
| Simonds, 2022 | 🗸 | 🗸 |  |  |  |  |  | * | 🗸 | 🗸 | * | * |  |  |  |  |  |  |  |  |  |  |  |  |  |  |  |

Note: 🗸 Yes X No *Can’t tell

Only four studies were considered to satisfy all quality criteria for quantitative non-randomized studies (Hartman et al., 1994; Lutze et al., 2014; Shoham et al., 2021; Willison et al., 2010). Nearly all study samples were representative of the target population, and all studies include measurements appropriate to the intervention and outcome. Studies were mixed in terms of the completeness of outcome data, and while one study did not account for confounders (Morley, 2016), in seven studies it was not clear whether confounding had been accounted for (Calathes, 1991; Davies & O’Meara, 2018; Elison et al., 2016; Hamilton & Campbell, 2014; Hignite & Haff, 2017; Twill et al., 1998; Vigesaa et al., 2016).

Based on MMAT criteria, all qualitative studies met all quality appraisal criteria. Similarly, both quantitative descriptive studies met all five quality appraisal criteria.

One mixed methods study met all five critical appraisal criteria (Williams, 2003). Of the remaining three studies, divergences and inconsistencies between quantitative and qualitative results were not clearly addressed (Lowenkamp & Latessa, 2002; Techagaisiyavanit et al., 2021; Yeboah, 2000). Two of these studies also did not make clear whether the different components of the studies adhered to the quality criteria of the methods involved.

## References

Boon, M. H., & Thomson, H. (2021). The effect direction plot revisited: Application of the 2019 Cochrane Handbook guidance on alternative synthesis methods. *Research Synthesis Methods*, *12*(1), 29–33. https://doi.org/10.1002/jrsm.1458

Calathes, W. (1991). Project Green Hope, a Halfway House for Women Offenders: Where Do They Go From Here? *Journal of Contemporary Criminal Justice*, *7*(2), 135–145. Scopus. https://doi.org/10.1177/104398629100700207

Davies, J., & O’Meara, A. (2018). Routine practice in staffed community accommodation (approved premises) in England and Wales: Quantitative benchmarking from the first year of a longitudinal study. *Criminal Behaviour and Mental Health*, *28*(3), 227–238. Scopus. https://doi.org/10.1002/cbm.2063

Elison, S., Weston, S., Davies, G., Dugdale, S., & Ward, J. (2016). Findings from mixed-methods feasibility and effectiveness evaluations of the “Breaking Free Online” treatment and recovery programme for substance misuse in prisons. *Drugs: Education, Prevention and Policy*, *23*(2), 176–185. Scopus. https://doi.org/10.3109/09687637.2015.1090397

Hamilton, Z. K., & Campbell, C. M. (2014). Uncommonly Observed: The Impact of New Jersey’s Halfway House System. *Criminal Justice and Behavior*, *41*(11), 1354–1375. Scopus. https://doi.org/10.1177/0093854814546132

Hartman, D. J., Friday, P. C., & Minor, K. I. (1994). Residential probation: A seven-year follow-up study of halfway house discharges. *Journal of Criminal Justice*, *22*(6), 503–515. Scopus. https://doi.org/10.1016/0047-2352(94)90092-2

Hignite, L. R., & Haff, D. R. (2017). Rapid rehousing of formerly homeless jail and prison inmates. *Housing, Care and Support*, *20*(4), 137–151. Scopus. https://doi.org/10.1108/HCS-06-2017-0015

Lowenkamp, C., & Latessa, E. (2002). *Evaluation of Ohio’s Community-Based Correctional Facilities and Halfway House Programs*.

Lutze, F. E., Rosky, J. W., & Hamilton, Z. K. (2014). Homelessness and Reentry: A Multisite Outcome Evaluation of Washington State’s Reentry Housing Program for High Risk Offenders. *Criminal Justice and Behavior*, *41*(4), 471–491. Scopus. https://doi.org/10.1177/0093854813510164

Morley, C. (2016). The relationship of personality characteristics and length of stay in a halfway house for previously incarcerated males. *Dissertation Abstracts International: Section B: The Sciences and Engineering*, *77*(Personality Psychology [3100]), No-Specified.

Schünemann, H. J., Higgins, J. P., Vist, G. E., Glasziou, P., Akl, E. A., Skoetz, N., Guyatt, G. H., & on behalf of the Cochrane GRADEing Methods Group (formerly Applicability and Recommendations Methods Group) and the Cochrane Statistical Methods Group. (2019). Completing ‘Summary of findings’ tables and grading the certainty of the evidence. In *Cochrane Handbook for Systematic Reviews of Interventions* (pp. 375–402). John Wiley & Sons, Ltd. https://doi.org/10.1002/9781119536604.ch14

Shoham, E., Efodi, R., Haviv, N., & Gross Shader, C. (2021). Dropout from Treatment and Desistance from Crime among Released Prisoners in Jerusalem Halfway House for Prisoners with Substance Misuse Disorder. *International Journal of Offender Therapy and Comparative Criminology*, 0306624X211010291. https://doi.org/10.1177/0306624X211010291

Techagaisiyavanit, W., Chokprajakchat, S., & Iyavarakul, T. (2021). How a halfway house became a road toward freedom: A case study of the Kalatapae community. *Journal of Criminological Research Policy and Practice*. https://doi.org/10.1108/JCRPP-01-2021-0002

Twill, S. E., Nackeruo, L., Riser, E. A., Eernat, J. A., & Taylor, D. (1998). Changes in measured loneliness, control, and social support among parolees in a halfway house. *Journal of Offender Rehabilitation*, *27*(3–4), 77–92. Scopus. https://doi.org/10.1300/J076v27n03_06

Vigesaa, L. E., Bergseth, K. J., & Richardson Jens, K. (2016). Who participates in reentry programming? An examination of women offenders in a midwestern state. *Journal of Offender Rehabilitation*, *55*(5), 308–328. Scopus. https://doi.org/10.1080/10509674.2016.1181131

Williams, D. J. (2003). “Quality of Life” as perceived by sex offenders on early release in a halfway house: Implications for treatment. *Journal of Offender Rehabilitation*, *38*(2), 77–93. Scopus. https://doi.org/10.1300/J076v38n02_05

Willison, J. B., Roman, C. G., Wolff, A., Correa, V., & Knight, C. R. (2010). *Evaluation of the Ridge House Residential Program: Final Report*.

Yeboah, D. (2000). The evaluation of New Zealand’s Habilitation Centre’s Pilot Programme. *JOURNAL OF CRIMINAL JUSTICE*, *28*(3), 227–235. https://doi.org/10.1016/S0047-2352(00)00038-6
